# Methodology for generating chorioallantoic membrane patient-derived xenograft (CAM-PDX) models of pleural mesothelioma and performing preclinical imaging for the translation of cancer studies and drug screening.

**DOI:** 10.12688/f1000research.163596.1

**Published:** 2025-05-07

**Authors:** Jan Schulze, Sarah Barnett, Liam Shaw, Anne Herrmann, Harish Poptani, Doris M Rassl, Alexander Haragan, Michael Shackcloth, Joseph J Sacco, Judy M Coulson

**Affiliations:** 1 Molecular and Clinical Cancer Medicine, University of Liverpool Institute of Systems Molecular and Integrative Biology, Liverpool, England, UK; 2 Egg Facility, Liverpool Shared Research Facilities, University of Liverpool Faculty of Health and Life Sciences, Liverpool, England, UK; 3 Centre for Preclinical Imaging, Liverpool Shared Research Facilities, University of Liverpool Faculty of Health and Life Sciences, Liverpool, England, UK; 4 Royal Papworth Hospital NHS Foundation Trust, Cambridge, England, UK; 5 Pathology Department, Royal Liverpool University Hospital, Liverpool, England, UK; 6 Liverpool Heart and Chest Hospital NHS Foundation Trust, Liverpool, England, UK; 7 Clatterbridge Cancer Centre NHS Foundation Trust, Bebington, England, UK

**Keywords:** Chorioallantoic membrane, PDX, pleural mesothelioma, 3Rs, preclinical model, PET/CT imaging, histology, immunohistochemistry

## Abstract

**Background:**

Pleural mesothelioma is a cancer of the lung lining associated with asbestos exposure. Platinum/pemetrexed chemotherapy has been used for many years but provides little benefit and, despite recent immunotherapy advances, prognosis remains poor underpinning the need for development of novel therapeutics or drug repurposing. Fertilized hens’ eggs provide a rapid and cost-effective alternative to murine models of pleural mesothelioma which are commonly used in preclinical studies, with chorioallantoic membrane (CAM) xenografts being a partial replacement for mouse flank xenografts. Here we describe methods to generate mesothelioma patient-derived xenografts on the CAM (CAM-PDX), and to subsequently assess these PDX nodules by preclinical imaging and histology.

**Methods:**

Fragments of surplus mesothelioma tissue obtained from patient biopsies were implanted onto the CAM on embryonic day 7 (E7), fresh or following cryopreservation, with the established PDX dissected on E14 and fixed for histological/immunohistochemical analysis. The optimal freezing method was determined by comparing tissue integrity and cellular content of cryopreserved tissue fragments with paired fresh samples via histological/immunohistochemical analyses. [ ^18^F]-fluorodeoxyglucose positron emission tomography/computed tomography (FDG-PET/CT) was used to assess viability of PDXs *in ovo.*

**Results:**

Methodologies for processing, cryopreservation, re-animation, and engraftment of mesothelioma tissue fragments were established. Cryopreservation of biopsy samples and parallel processing of contiguous sections allows for assessment of mesothelioma cellularity. CAM-PDXs, generated from fresh or slow-frozen tissue, were well vascularized whilst maintaining the architecture and cellular composition of the patient tissue. Furthermore, uptake of [ ^18^F]-FDG following intravenous injection could be visualized and quantified.

**Conclusions:**

The CAM is a rapid platform for engrafting patient-derived tissue, maintaining elements of the tumor microenvironment and recapitulating heterogeneity observed in mesothelioma. Combining the CAM-PDX model and FDG-PET/CT provides a quantitative *in vivo* platform for pre-screening of novel treatment strategies and drug combinations, with the potential for development of patient tumor avatars for predicting clinical response.

Research highlights **Scientific benefit(s)**

• Alternative preclinical model for translation of *in vitro* studies
• Tissue processing pipeline maximizes tumor cell content for efficient patient-derived model generation from mesothelioma biopsies
• Maintains tissue architecture and tumor microenvironment with host-derived vascularization
• Host-derived vascularization enables systemic drug delivery and radiotracer administration to monitor viability

**3Rs benefit(s)**

• Direct replacement for mouse PDX models
• The chick embryo model can be used as a pre-screening platform before moving into protected mammalian models (reduction)
• Facilitates drug testing without, or minimizing, the use of mammalian models

**Practical benefit(s)**

• Scalable, low cost and rapid preclinical model
• Facilitates *in vivo* preclinical testing in a higher throughput manner than mammalian models
• Brings together scientific and clinical teams
• Amenable to repurposing of small animal imaging modalities

**Current applications**

• In our lab, the CAM-PDX model in combination with PET/CT imaging is being utilized for the assessment of novel drug combinations and therapeutic strategies for improved treatment responses in mesothelioma

**Potential applications**

• Preclinical screening for novel drug combinations and therapeutic strategies, patient stratification and repurposing of drugs for other cancers
• Preclinical evaluation of patient suitability for clinical trials
• Development of rapid real-time treatment response (patient tumor avatars)

## Introduction

Pleural mesothelioma is a cancer of the lining of the lungs that is predominantly caused by asbestos exposure. Most cases can be histologically classified as either epithelioid, sarcomatoid or biphasic ( Lokuhetty *et al.*, 2021), with loss of tumor suppressors being a common genetic feature. For example, loss or inactivation of the nuclear deubiquitylase BRAC1-associated protein (BAP1) is found in 60-70% of cases and is most frequent in the epithelioid subtype ( Bott *et al.*, 2011). Despite the recent approval of immunotherapy for untreated mesothelioma ( Baas *et al.*, 2021), pleural mesothelioma remains an incurable disease with poor prognosis. There is, therefore, still a pressing need for new therapies, and consequently the requirement for drug screening and preclinical testing. Typically, rodent models would be used for such studies; however, these models are costly, labor-intensive to maintain, take months to produce results ( Reyal *et al.*, 2012), and there is a growing demand to reduce the use of protected animals in research. Despite a declining trend in the use of mice since 2007, they remain a commonly used model with 60% of all animal experiments in 2023 being conducted in this species, which equates to around 0.9 million mice in the UK alone ( Baxter, 2024). Around one-fifth of these animals were used for either basic or translational research of human cancer and 40% of all mouse experiments involved non-genetically modified models ( Baxter, 2024), such as xenografts. If a fraction of these were replaced with non-mammalian models it would save thousands of mice per year.

The extra-embryonic chorioallantoic membrane (CAM) of fertilized hens’ eggs can be used to generate cancer xenografts for the translation of *in vitro* cancer studies. The CAM model has multiple benefits; being cheaper and having fewer husbandry requirements compared to mammalian models. It is also an attractive option as avian embryos are non-protected up to two thirds gestation, with pain perception only becoming functional from E15 onwards in chickens ( Süß *et al.*, 2023), and so they do not fall under Home Office regulations up to embryonic day 14 (E14) in the UK. Thus, the CAM is both more ethical and simpler for researchers to adopt as an *in vivo* model than mice. This 3Rs-compliant model has been successfully employed to study the key hallmarks of cancer, and for evaluation of new therapeutics, and thus can significantly contribute to reducing or replacing use of rodent models in cancer research ( Schneider-Stock and Ribatti, 2021).

We have previously shown the utility of the CAM model for the generation of mesothelioma cell line xenografts ( Barnett *et al.*, 2022). While cell lines are a tractable option and are useful for optimizing protocols and experimental workflows, patient-derived models are more representative. The recent development of a breadth of *in vitro* patient-derived models for mesothelioma, such as organoids ( Shamseddin *et al.*, 2021) and explants ( Powley *et al.*, 2020), is important for driving forward mesothelioma research. However, the choice of model should be carefully considered. For instance, patient-derived organoids (PDO) lose the original tissue architecture and cellular composition, whereas both patient-derived explants (PDE) and patient-derived xenografts (PDX) retain these features. Since mesothelioma is a highly fibrotic cancer, with stromal and immune cells influencing tumor progression, maintaining the tumor microenvironment is particularly important to ensure a more representative treatment response ( Chrisochoidou *et al.*, 2023). Furthermore, a key benefit of CAM xenografts over both explants and organoids is the vascularization of tumor nodules, which maintains viability, whereas explants frequently become necrotic. This vascularization also enables systemic delivery of therapies and reagents through chick-derived blood vessels, via intravenous or yolk-sac injection, thus offering a more physiologically relevant means of treatment administration.

Although, several mouse models of mesothelioma have been developed including asbestos-induced ( Grosso *et al.*, 2021), conditional genetic knockouts ( Kukuyan *et al.*, 2019) and, of most relevance here, xenograft or PDX models ( Wu *et al.*, 2017), these can manifest severe phenotypes. As well as ethical considerations, establishing PDXs in mice can be difficult, with a take rate of only 40-50% in some cases ( Boughey *et al.*, 2021) and mesothelioma is no exception ( Wu *et al.*, 2017). Furthermore, generating mouse PDX models can take several months, with reports of 2-8 months for first generation engraftment in breast cancer for example ( Reyal *et al.*, 2012). Thus, for applications such as patient avatars, this may be futile in mesothelioma where median survival is 12-21 months, and only 7 months for the sarcomatoid subtype ( Amin *et al.*, 2018). The CAM xenograft assay can be considered as a direct replacement for mouse xenograft flank models and has been tested as a PDX platform for a variety of other cancer types such as glioblastoma ( Shoin *et al.*, 1991), clear cell renal cell carcinoma ( Fergelot *et al.*, 2013), as well as one publication reporting use of mesothelioma tissue ( Mîndrilă *et al.*, 2017). Published studies report high engraftment rates (70-80%), histological concordance with tumor type and a strong correlation between CAM-PDX assay results and patient outcome ( Shoin *et al.*, 1991, DeBord *et al.*, 2018, Charbonneau *et al.*, 2023), supporting the CAM model as an alternative method for generating physiologically applicable PDXs that can be utilized for drug screening and personalized medicine.

PET/CT is routinely used in the clinic for detection and staging of cancer, mesothelioma included, as well as monitoring of treatment response in patients ( Kruse *et al.*, 2013). Moreover, since mesothelioma has a large stromal compartment, an imaging technique that selectively reports on cancer cell metabolism as a surrogate marker for viability may be more informative of treatment response than standard readouts such as tumor weight. Additionally, as others have previously demonstrated the feasibility of applying PET/CT imaging to cell line-derived CAM xenografts this was our modality of choice ( Warnock *et al.*, 2013, Smith *et al.*, 2023, Schulze *et al.*, 2023a). Hence, we developed a workflow to combine CAM-PDX and PET/CT imaging to facilitate assessment of treatment response in this model.

To make this approach accessible to other laboratories as a replacement for mouse PDXs, our aim was to develop robust protocols that describe in detail how to generate CAM-PDX models of mesothelioma and monitor tumor cell composition and health to assess therapeutic responses. Our specific objectives were to: (1) Optimize a pipeline for processing, characterization and cryopreservation of biopsy samples to ensure availability of high-quality tissue for engraftment; (2) Demonstrate robust engraftment of tissue, and preservation of architecture and cellular composition in PDX nodules; and (3) Apply *in ovo* [ ^18^F]-FDG PET/CT imaging to measure cancer cell metabolism within PDX nodules. These CAM-PDX methods can be extrapolated to other tumor types, and the tissue processing method could be applied to the generation of PDEs, or even mouse PDX models, to increase experimental efficiency thus having a wider impact across models.

## Materials

Supplier information and catalogue numbers for reagents, consumables and equipment are detailed in Table 1.

**Table 1.** Materials used.

Item	Brand/Supplier	Catalogue number (Batch Number)
10% Neutral buffered formalin (NBF)	Sigma	HT501128-4L (Lot MKCG3336)
Epredia™ Cassette II Biopsy Tissue Cassettes	Fisher Scientific	16324136
Ethanol	Fisher Scientific	From university chemistry department
Xylene (processor)	Fisher Scientific	10385910
Surgipath formula R paraffin wax	Leica	3801470
MX35 Ultra microtrome blades (3053835)	SLS	HIS3050
Superfrost plus adhesion slides	VWR	631-9483
Harris Hematoxylin (H&E autostainer)	Leica	3801560BBE
Eosin Y (H&E autostainer)	Leica	3801600BBE
Pertex mounting media (Histology coverslipper)	Leica	3808706E
2450-1.0 Micro Coverglass (Histology coverslipper)	Leica	3800145G
BOND Dewax Solution	Leica	AR9222
BOND Wash Solution	Leica	AR9590
BOND Epitope Retrieval Solution 2	Leica	AR9640
BOND Primary Antibody diluent	Leica	AR9352
BOND Polymer Refine Detection Kit - Peroxide Block (30 mL) 3–4% (v/v) Hydrogen peroxide - Post Primary (30 mL) Rabbit anti mouse IgG (<10 μg/mL) in 10% (v/v) animal serum in tris-buffered saline/0.1% ProClin™ 950 - Polymer (30 mL) Anti-rabbit Poly-HRP-IgG (<25 μg/mL) containing 10% (v/v) animal serum in tris-buffered saline/0.1% ProClin™ 950 - DAB Part 1 (2.4 mL) 66 mM 3,3’-Diaminobenzidine tetrahydrochloride hydrate, in a stabilizer solution - DAB Part B (30 mL) ≤0.1% (v/v) Hydrogen Peroxide in a stabilizer solution - Hematoxylin (30 mL) <0.1% Hematoxylin	Leica	DS9800
Xylene (Immunohistochemistry)	VWR	28975.325
DPX mountant for histology (Immunohistochemistry)	Sigma	#06522
22 × 50 mm glass coverslips - thickness #1	VWR	6310137
2ml cryovials	Corning	430488
Mr. Frosty™ Freezing Container	Thermo Fisher Scientific	5100-0001
No.11 Disposable scalpels	Swann Morton	#0503
Gibco10X Dulbecco's phosphate-buffered saline	Thermo Fisher Scientific	70011036
Fetal Bovine Serum (FBS)	Gibco	10437-028 (Lot 2405703RP) A5256701 (Lot 2575628)
RPMI 1640 Glutamax	Gibco	61870-010 (Lot 2436534)
Dimethyl sulfoxide (DMSO)	Sigma	D8418
Gibco 100X Penicillin-Streptomycin (10000 U/mL)	Fisher Scientific	11548876
1.5 mL TubeOne Microcentrifuge Tube	Starlab	S1615-5500
Corning 50 mL non-skirted Tubes	Appleton Woods	BC034
10cm plates	Starlab	CC7682-3394
Corning 35 mm untreated cell culture dishes	Appleton Woods	BC146
Egg chiller	Haier	WS46GDBE
Brinsea OvaEasy 380 Advance EX Series II Automatic Egg Incubator	Brinsea	MJ3833A
Brinsea Disinfectant	Brinsea	14.35
Egg piercer	Amazon	B09VYQHCMV
Scotch Magic Tape 810 Solvent-Free Transparent 25mmx66m	Banner	3913933
Orange Labeling Tape (0.5 inch-wide x 500 inch)	SLS	TAP1088
Needles 20g 1inch (0.9x25mm)	VWR	613-5391
5mL syringe without needle 6% leur (SS05SE)	SLS	SYR6204
Lens cleaning tissue (Lens tissue)	Fisher Scientific	10166284
Portex Tubing medical and surgical PVC 4mm diameter	Fisher Scientific	13130863
Kimtech precision wipes	Kimberley Clark	7552
Meso-relle 33Gx1/2” 0.2x12mm needle	UKMEDI	AM33G
BD - PlastiPak Luer Slip Syringe without Needle 1 mL	BD	303172
NaCl 0.9% (0.9% w/v Sodium Chloride Injection BP)	B.Braun	2350756

## Methods

### Patient biopsy tissue processing and cryopreservation

**Sample collection**

Fresh surplus biopsy tissue samples were donated under written informed consent from treatment-naïve patients undergoing diagnostic percutaneous biopsies at NHS Liverpool Heart and Chest Hospital Teaching Hospitals between March 2021 and November 2024. All specimen collection was conducted under the approved ethics (East of England – Cambridge Central Research Ethics Committee; approved 9 ^th^ August 2019, 18/EE/0161; renewed 30 ^th^ August 2023, 23/EE/0139) for the mesothelioma biobank, Mesobank UK ( Rintoul *et al.*, 2016). Samples were immediately placed into a 50 mL centrifuge tube containing 25 mL RPMI 1640 Glutamax (Thermo Fisher Scientific, MA, USA) supplemented with 1% penicillin-streptomycin (Fisher Scientific, NH, USA) and kept on ice. Samples were assigned a unique Mesobank sample identifier and transferred on ice to the research laboratory (University of Liverpool) within 30 minutes. All experiments were conducted according to the Declaration of Helsinki and in compliance with all local policies and standard operating procedures for working with human material.

**Preparation of tissue samples for implantation, freezing or fixation for histology**

**Figure 1.**
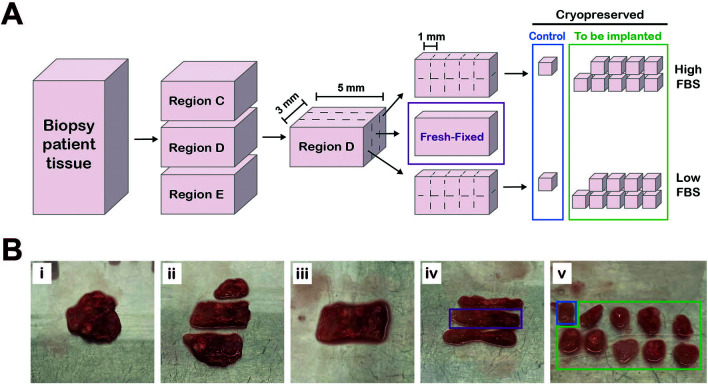
Preparation of patient tissue for parallel histological assessment and cryopreservation. A, Workflow diagram of how tissue was processed. Figure created in Adobe Illustrator 2025 (Version 29.4 (64-bit), RRID:SCR_010279). B, Series of photographs showing tissue preparation. Pleural biopsy tissue (Bi) cut into different regions (Bii). A single region (Biii) cut into three equally sized sections (Biv). The middle section (purple box, “fresh-fixed”) was fixed in 10% NBF and the outer sections were cut into 1 mm ^3^ fragments (Bv) and slow-frozen in either high FBS or low FBS cryopreservation media ( Table 2). One piece from each section (blue box) served as a control for the freezing process.

All work with human tissue was carried out in a ESCO Airstream class II biosafety cabinet (ESCO Lifesciences Group, Singapore) and all equipment (10 cm dishes, tweezers, scalpel) were sterilized via ultraviolet (UV) light prior to collecting the sample. Immediately upon arrival at the lab, the contents of the 50 ml tube were transferred into a 10 cm plate (Starlab, UK). The sample was then immediately cut into 1 mm ^3^ fragments with a scalpel (Swann Morton, UK) according to the diagram in Figure 1. Tissue pieces were kept in transport media to prevent the tissue drying out.

**Cryopreservation of tissue**

Two milliliter cryovials (Corning, NY, USA) were prepared with 1 mL freezing media ( Table 2) immediately prior to collection of samples, or ahead of time and frozen at -20°C. Using tweezers, a maximum of up to five 1 mm ^3^ fragments were transferred into one cryovial ( Figure 1). Vials were placed into a Mr. Frosty freezing container (Thermo Scientific; MA, USA) and transferred to a -80°C freezer (Sanyo Electric Company Ltd., Japan) as quickly as possible. Samples were then transferred to liquid nitrogen (vapor phase) for long-term storage within a week of processing.

**Table 2.** Freezing media components (% v/v).

Component	High FBS	Low FBS
RPMI 1640 Glutamax	0	80
Fetal Bovine Serum (FBS)	90	10
Dimethyl sulfoxide (DMSO)	10	10

**Reanimation of cryopreserved tissue**

Frozen vials were transported on dry ice and placed in a water bath (Grant Instruments Ltd, UK) at 37°C for 30-60 seconds. Vials were agitated until the contents had thawed, ensuring all ice crystals had melted. The contents of the cryovial were poured into a 10 cm plate, ensuring no fragments were stuck in the tube. The tissue fragments were thoroughly rinsed to remove all cryoprotectant solvent by adding 5 ml of sterile Dulbecco’s phosphate-buffered saline (PBS; Thermo Scientific) to the dish and aspirating carefully. This was repeated two times prior to proceeding with CAM implantation or fixation for histology.

**Chorioallantoic membrane (CAM) assay**

Our methods for generating cell-line derived xenografts have briefly been described previously ( Barnett *et al.*, 2022), some stages of the process are very similar but here we provide a more detailed overview.

**Egg and incubator information**

**Figure 2.**
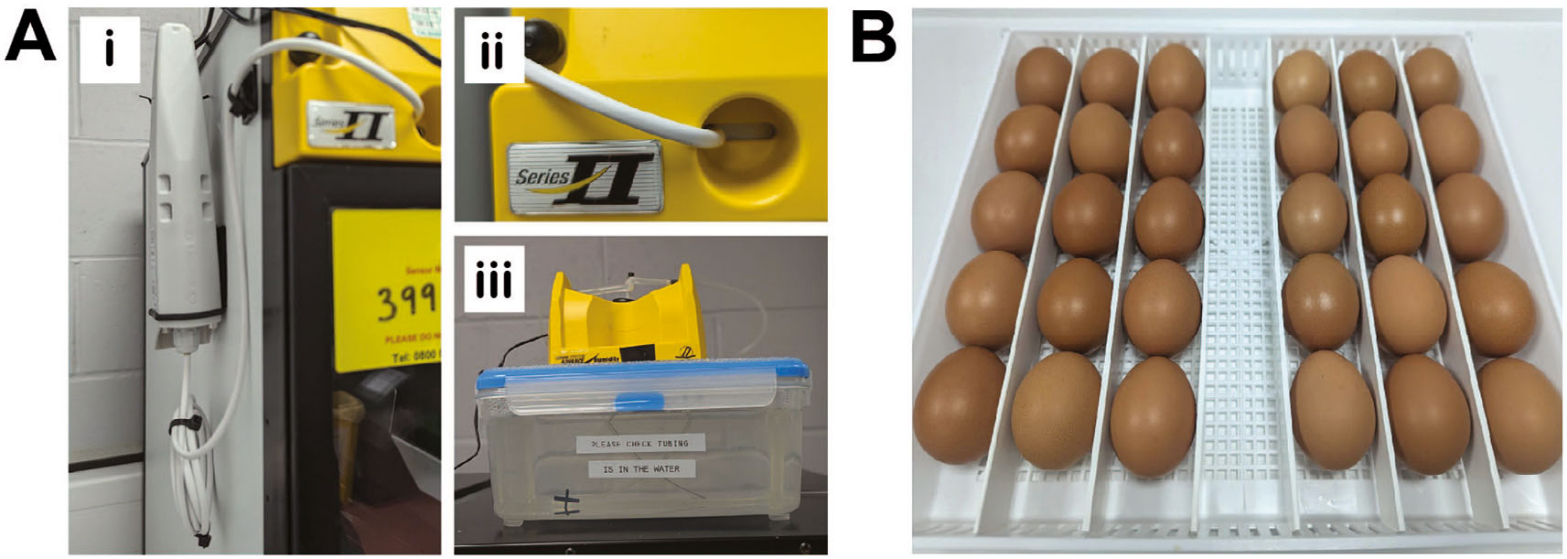
Technical considerations for egg experiments. A, Brinsea egg incubator modifications. Dual humidity and temperature T-Scan probes are fitted inside the egg incubators via the front vent (Ai-ii). 5L plastic containers are used as water reservoirs to increase the water capacity (Aiii). B, Eggs are incubated horizontally and placed to ensure they are touching neighboring eggs and unable to roll.

Fertilized brown chicken eggs were purchased from MedEggs Ltd (UK) and stored for a maximum of 2 weeks at 14°C in a temperature-controlled chiller (Haier, China) until starting the experiments. Egg incubation was carried out in OvaEasy 380 Advance EX Series II Automatic egg incubators fitted with humidity pumps (Brinsea, UK) ( Figure 2A). Temperature and humidity were set at 37.8°C and 50%, respectively and continually monitored via dual humidity and temperature probes (T-Scan Solutions, UK).

**Initiating embryo development**

Embryonic development was initiated by transferring eggs to 37°C. Egg trays and dividers were cleaned with 1X Brinsea disinfectant prior to adding the eggs. Eggs were cleaned with 1X Brinsea disinfectant by spraying the solution onto 2-ply blue roll and then thoroughly wiping each egg, to ensure all contaminant and foreign bodies were removed. The eggs were placed on their side in a row (max 5 per row, maximum 30 per tray), starting at one end of the tray ensuring that each egg was butted up against the next one to prevent eggs rotating forwards ( Figure 2B). Each egg was labelled with pencil to indicate start date and incubated with rocking (shelf rotation set to 45°, rotating every 45 minutes) from embryonic day 0 (E0) until E3.

**Windowing**

On E3, eggs were taken one at a time from the incubator and a small window was cut in the eggshell to provide access to the CAM. To allow for the opening to be cut, a second air pocket was made by pricking a hole in the blunt end of the egg (location of the natural air cell) with an egg piercer (Amazon, WA, USA). Next, a 5 mL syringe (Scientific Lab Supplies Ltd, UK) equipped with a 20 g 1 inch needle (Fisher Scientific) was inserted through the hole at a 45° angle to the bottom of the shell and 3-5 mL of albumin was extracted. The hole was sealed with a small square piece of orange tape (Scientific Lab Supplies Ltd). Next a three-sided rectangular window (approximately 2 cm × 1 cm) was cut in the top of the egg (the side facing up during E0-E3 incubation). Firstly, a hole where the scissors were to be inserted to cut the window was created with the egg piecer, then a piece of Scotch Magic Tape (Banner, UK) was placed over the region to be cut to prevent eggshell falling into the egg. Next, the point of the scissors was inserted into the pre-made hole and the rectangle cut, ensuring scissors were kept horizontal to avoid damage to the embryo. The egg was visually inspected for a viable embryo (determined via observation of a beating heart) and then the window sealed with a second piece of Scotch tape. Eggs were then incubated as before but without rocking until the end of the experiment.

**Implantation**

In preparation for implantation: lens tissue (Fisher Scientific) was cut into 1 cm × 2 cm strips and UV sterilized for 1 hour; 1-2 mm thick rings for implanting were cut from Portex tubing (1 mm wall thickness; 4 mm inner diameter) and sterilized overnight in 70% ethanol; and tweezers were cleaned (1 for lens tissue, 1 for opening egg window, 1 for handling tissue fragments). On E7 eggs were inspected to ascertain how many viable eggs were available for implant, and any non-viable eggs were discarded. In case of any leakage from the egg, a new tray lined with 2-ply blue roll was prepared. The eggs were returned to 37°C until ready to implant. The required number of vials of tissue were thawed (see section on reanimation of cryopreserved tissue fragments). One egg was removed from the incubator at a time for implantation to reduce temperature fluctuations. The piece of eggshell covering the window was removed completely, transferred to a new piece of scotch tape and left facing upwards in the hood. The egg was inspected to locate the CAM and identify an appropriate region for implantation, ideally at the bifurcation of a blood vessel. Then, using sterile tweezers, a strip of folded lens tissue was used to touch the CAM to dry/traumatize the area to be implanted. This was repeated until the area was sufficiently dry (indicated by the tissue sticking to the CAM) or a small bleed was visible. One sterilized ring was then placed on the area and one tissue fragment placed inside it. The window was resealed with the tape holding the piece of eggshell, with a second piece added perpendicularly to form a cross and ensure the window was fully sealed to reduce evaporation. The egg was given a unique identifier, labelling with pen on the colored tape on the blunt end or on the eggshell in pencil, and carefully transferred back to the incubator.

**Dissection and Imaging of CAM-PDX**

On E14, CAM-PDXs were imaged and dissected. PET/CT may be performed on E13 (see *In ovo* PET/CT imaging section), and where this was the case, the residual radioactivity was measured with a radiation monitor (Radhound; Southern Scientific, UK) to ensure the radiotracer has decayed sufficiently (below 1000 count per second) prior to dissection. Before starting, 1 ml aliquots of 10% neutral buffered formalin (NBF) were prepared in 1.5 ml microfuge tubes (1 per sample) in a fume hood. For imaging *in situ*, the egg was placed in a holder, such as a 35 mm dish, the window in the eggshell was enlarged (if not done at a previous stage) and brightfield images of the xenograft on the CAM were captured using Leica M165FC fluorescence stereomicroscope with 16.5:1 zoom optics, fitted with a Leica DFC425 C camera (Leica Biosystems, Germany). Then the PDX was excised. This was achieved using tweezers, such as No.5 110 mm tweezers (Dumont, Switzerland), to hold the CAM taut and cutting around the PDX with micro-spring scissors (Fine Science Tools, Germany) ensuring 0.5-1.0 cm of CAM remained around the sample. The sample was placed in a drop of PBS in a 10 cm dish and the embryo immediately terminated via decapitation *in situ.* The sample was washed briefly to remove excess blood by agitation in the PBS droplet and then transferred to a fresh drop of PBS for imaging. The sample was orientated using two pairs of tweezers so that the underneath of the sample could be imaged, whilst ensuring the CAM was laid out flat. The sample was then placed into fixative.

**Passaging**

On E14 the resulting CAM-PDX was dissected as described in the previous section. Following imaging, the PDX was trimmed using micro-spring scissors to remove the CAM and then cut in half using a sterile scalpel. One half was then implanted onto the CAM in an E7 egg, as described above, ensuring that the freshly cut side was placed onto the prepared CAM.

**CAM-PDX scoring**

**Figure 3.**
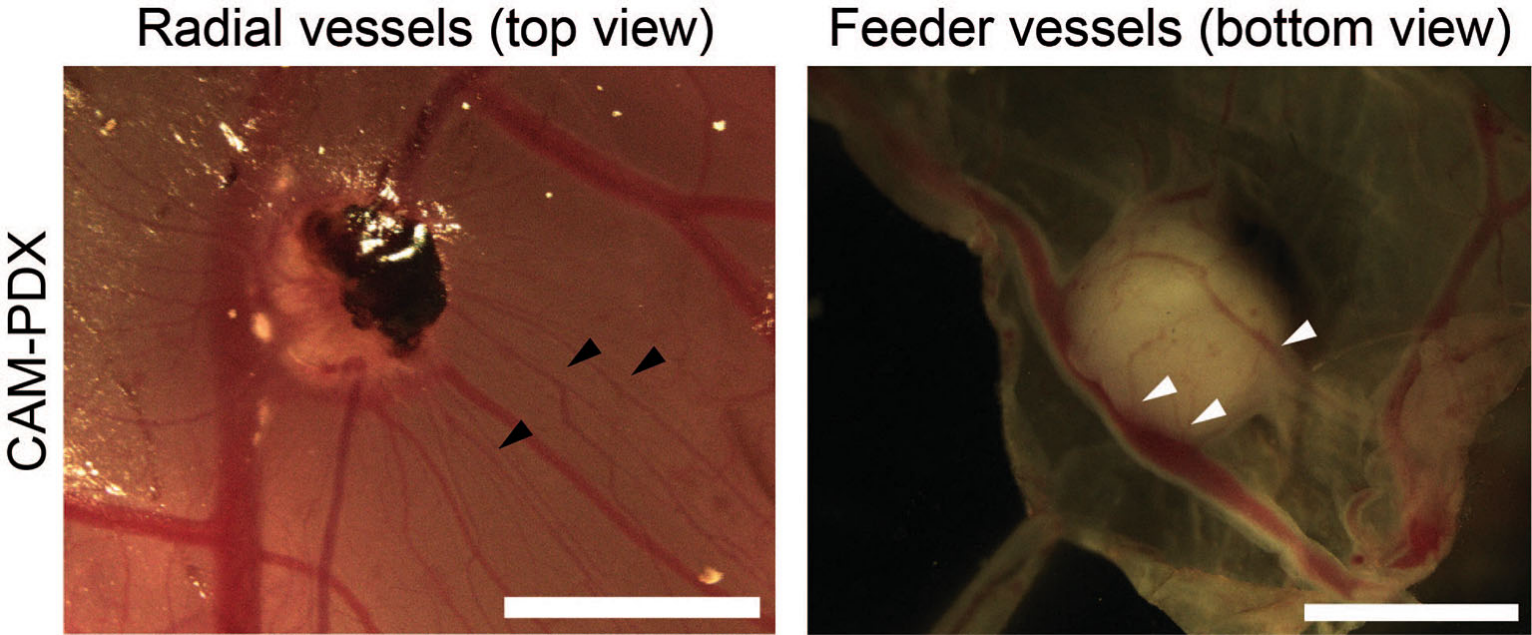
Scoring CAM-PDX for vascularization as a surrogate marker of engraftment. Representative images of radial vessels (black arrowheads) surrounding the CAM-PDX in ovo and feeder vessels (white arrowheads) visible on the underneath of the CAM-PDX post-dissection. Scale bar represents 2.5 mm.

Each CAM-PDX was evaluated macroscopically for vascularization, as an indicator for engraftment, by assessing two images. Images of the PDX *in situ* on the CAM and post-dissection from the underneath were scored for the presence of radial and feeder vessels, respectively ( Figure 3). Scoring was conducted by two independent observers. Eggs were classified as ‘not scorable’ if a decision could not be rendered by both observers with example cases including inadequate image quality, the CAM-PDX being too close the eggshell or too much blood on the CAM.

### Histology

**Fixation, processing, embedding and sectioning**

**Figure 4.**
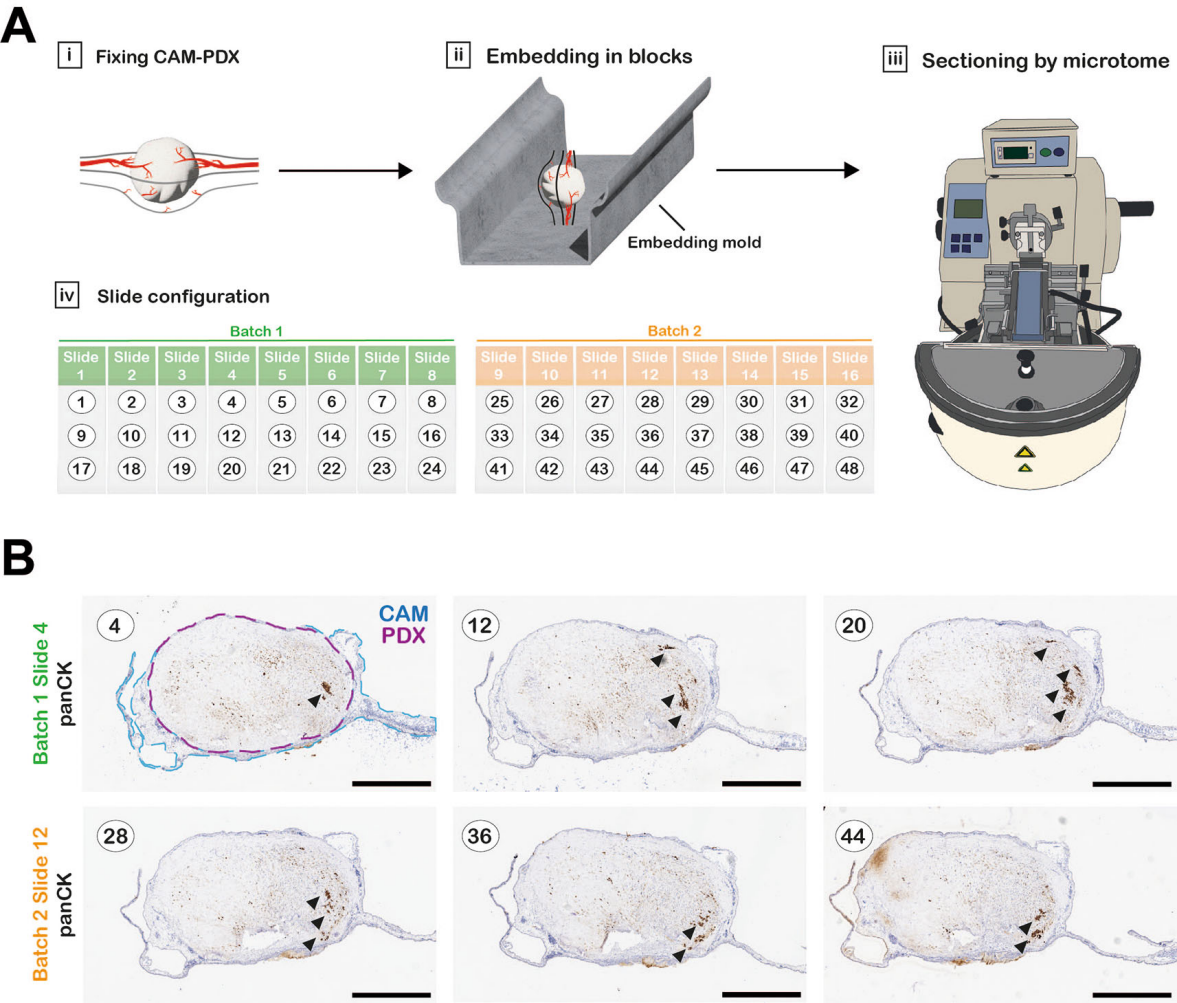
Technical considerations for histology. A, Workflow diagram of CAM-PDX processing for histology. CAM-PDX samples are embedded in paraffin so that sectioning the block results in sagittal sections through the tumor nodule and CAM (Ai-iii). Sections (here numbered 1-48 as an example) are placed onto slides in a configuration of 3 sections per slide that provides an overview through the xenograft with minimal slide staining; numbering (black text) refers to sequentially cut sections (Aiv). Sections are collected in up to 10 batches containing 8 slides that will be stained for different markers on contiguous slides. Figure created with Adobe Illustrator 2025 (Version 29.4 (64-bit), RRID:SCR_010279). B, One slide per batch was stained for pan-Cytokeratin to identify areas within the sample with detectable tumor cells (indicated by arrowheads), to identify the most suitable batch for staining for further markers. Scale bars represent 1000 μm.

Tissue fragments or CAM xenografts were placed in 1.5 mL tubes containing 1 mL 10% NBF and fixed for a minimum of 16 hours. The 10% NBF was removed and replaced with 1 mL 70% ethanol. Prior to processing, tissue samples were transferred to biopsy tissue cassettes (Fisher Scientific) and kept submerged in 70% ethanol until they were processed. Any small tissue fragments, such as “fresh-fixed” 1 mm ^3^ controls, were wrapped in lens tissue prior to placing inside the cassettes to prevent loss of the small samples during processing. The cassettes containing the samples were loaded into a Leica ASP 300S tissue processor and run according to the program outlined in Table 3. Upon completion, samples were immediately transferred to a Leica EG1150H embedding station and samples embedded in Formula R paraffin wax. CAM xenograft samples were embedded in an orientation to ensure a sagittal section through the sample ( Figure 4A). The paraffin blocks were sectioned using a Thermo HM 340E Electronic Rotary Microtome fitted with MX35 ultra blades. Four-micron sections were collected on SuperFrost plus adhesion slides (according to layout in Figure 4Aiv and 4B) and placed in an oven overnight at 37°C.

**Table 3.** Tissue processor program.

Reagent	Duration	Temperature	Pressure (P)/Vacuum (V)	Drain time (seconds)
Ethanol 70%	5 minutes	Room temperature	V	140
Ethanol 90%	10 minutes	Room temperature	V	140
Ethanol 100%	5 minutes	Room temperature	V	140
Ethanol 100%	5 minutes	Room temperature	V	140
Ethanol 100%	5 minutes	Room temperature	V	140
Ethanol 100%	10 minutes	Room temperature	V	140
Xylene	10 minutes	Room temperature	V	140
Xylene	10 minutes	Room temperature	V	140
Xylene	10 minutes	40°C	V	140
Paraffin Wax	5 minutes	62°C	V	140
Paraffin Wax	15 minutes	62°C	V	140

**Hematoxylin and Eosin (H&E) staining**

Automated H&E staining and cover slipping was carried out on a Leica ST5020-CV5030 Stainer Integrated Workstation according to program shown in Table 4.

**Table 4.** Hematoxylin and eosin (H&E) auto stainer program settings.

Reagent	Timing
Xylene	2 minutes
Xylene	2 minutes
100% Ethanol	1 minute
90% Ethanol	1 minute
70% Ethanol	1 minute
Water	1 minute
Hematoxylin (Harris)	5 minutes
Water	1 minute
1% Acid Alcohol	15 sec
Water	5 minutes
Eosin Y (alcoholic)	3 minutes
Water	15 seconds
70% Ethanol	30 seconds
90% Ethanol	30 seconds
100% Ethanol	30 seconds
Xylene	2 minutes
Xylene	2 minutes

**Immunohistochemistry**

Antibody staining was conducted on a Leica Bond RXm automated stainer. The staining program used ( Table 5) was the BOND Polymer Refine IHC Protocol, utilizing the BOND Polymer Refine Detection system which is supplied as ready-to-use containing a peroxide block, post primary (secondary antibodies: rabbit anti-mouse IgG and anti-rabbit Poly-HRP IgG), polymer reagent, 3,3’-Diaminobenzidine tetrahydrochloride hydrate (DAB) chromogen for visualization and hematoxylin counterstain. Details of primary antibodies are provided in Table 6. All antibodies were optimized in-house on appropriate control tissue and staining performed in parallel to negative or isotope controls ( Barnett *et al.*, 2022). At the end of the staining program, slides were removed and placed in a staining rack. The rack was placed in a slide-staining pot and rinsed continually for 5 minutes with cold tap water, ensuring that the flowing water did not run directly onto the slides. The slides were then passed through a dehydration series consisting of 3X washes in 100% ethanol and 2X washes in xylene. Slides were gently agitated during each wash step for 30 seconds. Slides were kept in the final xylene solution and removed one at a time for cover slipping. Each slide was sealed with DPX mountant and a single 22 × 50 mm coverslip, ensuring any bubbles were removed.

**Table 5.** Leica Bond RXm immunohistochemistry (IHC) staining program settings.

Step	Reagents	Time
Dewax	BOND Dewax Solution, 100% Alcohol, BOND Wash Solution	Pre-programmed Leica BOND
Antigen Retrieval	BOND Epitope Retrieval ER1 Solution or BOND Epitope Retrieval ER2 Solution	HIER 20 minutes with ER1 or HIER 20 minutes with ER2
WASH	BOND Wash Solution	3 × 0 minutes * 1 × 3 minutes
Peroxide Block	Refine Detection Kit Peroxide Block *	5 minutes
WASH	BOND Wash Solution	3 × 0 minutes
Primary Antibody	Diluted in #8112 SignalStain® Antibody Diluent	15 minutes
WASH	BOND Wash Solution	3 × 2 minutes
Secondary Detection	Rabbit anti mouse IgG <10 μg/ml in 10% v/v animal serum in Tris buffered saline/0.1% ProClin™ 950	8 minutes
	Anti-rabbit Poly-HRP-IgG <25 μg/ml in 10% v/v animal serum in Tris buffered saline/0.1% ProClin™ 950	8 minutes
WASH	BOND Wash Solution/Deionized Water	Custom (see main text)
Visualization	BOND Refine Detection Kit Mixed DAB *	10 minutes
WASH	Deionized Water	3 × 0 minutes
Counterstain	BOND Refine Detection Kit Hematoxylin *	5 minutes
WASH	Deionized Water	0 minutes
WASH	BOND Wash Solution	0 minutes
WASH	Deionized Water	0 minutes

* Wash steps set to 0 minutes indicates slides were flushed with no incubation.

**Table 6.** Antibodies used for immunohistochemistry.

Antibody	Clonality	Catalogue #	Batch No	Brand	RRID	Dilution	Antigen retrieval	Reactivity
Pan Cytokeratin (MNF116)	monoclonal	MA1-26237	XK3746097F	Invitrogen	AB_794730	1:80	ER2	Human
Calretinin (CALB2)	polyclonal	HPA007306	A83215	Sigma	AB_1078385	1:75	ER1	Human
BAP1 (C-4)	monoclonal	sc-28383	I2517	Santa Cruz Biotechnology	AB_626723	1:250	ER2	Human
Cleaved Caspase-3 (Asp175)	polyclonal	#9661	47	Cell Signaling Technology	AB_2341188	1:500	ER2	Human
Ki67	monoclonal	NCL-L-Ki67-MM1	6062741	Novocastra	AB_563841	1:200	ER2	Human
Alpha smooth muscle actin	polyclonal	ab5694	GR3432718-3	Abcam	AB_2223021	1:200	ER2	Human, chicken
CD4	monoclonal	NCL-L-CD4–368	46168	Novocastra	AB_563559	Ready to use	ER2	Human
CD8	monoclonal	M7103	20011401	Agilent Dako	AB_2075537	1:200	ER2	Human

**Slide scanning and image processing**

Whole slide scans were obtained using Leica Aperio CS2 digital slide scanner (services provided by Liverpool University Biobank). Images were viewed in QuPath (RRID:SCR_018257) ( Bankhead *et al.*, 2017) and scale bars added via the built-in ImageJ extension (RRID:SCR_003070) ( Schindelin *et al.*, 2012).

### *In Ovo* Positron-Emission-Tomography/Computed Tomography (PET/CT) imaging

**Radiotracer preparation**

**Figure 5.**
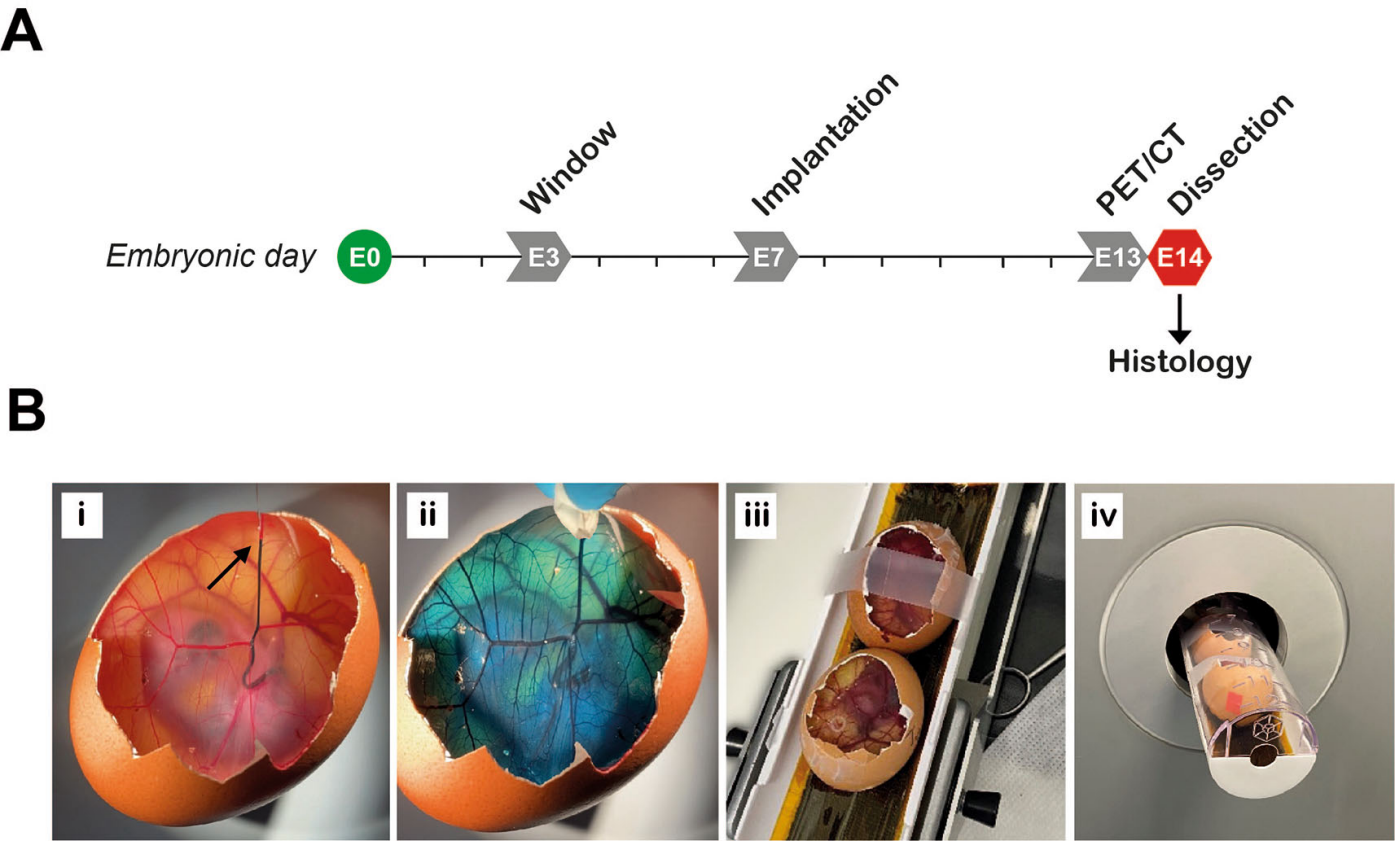
Preparation of eggs for preclinical PET/CT imaging. A, Experimental timeline. PET/CT performed on embryonic day 13 (E13). B, Preparing the eggs for imaging. Radiotracer is injected intravenously, demonstrated here by injecting food dye at the point indicated by the arrow (Bi). Approximately 30 seconds after injection, food dye was completely distributed within the egg (Bii). Immediate compression (a few seconds after injection) should be used to stop any bleeding at the site of injection if required. Two eggs are fixed in position in the imaging bed with tape (Biii) and then loaded into the PET/CT scanner (Biv).

PET/CT imaging of tumors may be performed *in ovo*, as optional analysis prior to dissection of the tumor nodules. Since the half-life of [ ^18^F]-FDG is approximately 110 minutes ( Ido *et al.*, 1978), samples are safe to handle the day after radiotracer injection, and PET/CT imaging can therefore be performed on E13 followed by dissection and termination of experiments on E14 ( Figure 5A). [ ^18^F]-FDG (1000 MBq; Alliance Medical Radiopharmacy, UK) was ordered for delivery on the morning of E13 for each experiment. Upon arrival total radioactivity was measured and typically ranged from 600-800 MBq. Behind lead shielding, the [ ^18^F]-FDG stock solution was diluted in a 1.5 mL microfuge tube with sterile 0.9% sodium chloride solution (B.Braun, Germany) to give approximately 60 MBq [ ^18^F]-FDG in 1.5 mL. Due to the relatively short half-life of [ ^18^F]-FDG, the tracer volume was adjusted each time the tracer was prepared for injection. The injected radioactivity per egg was 5 ± 1 MBq in a final volume of 100-150 μL, administered using a 1 mL BD Luer slip syringe (Scientific Laboratory Supplies Ltd, UK) with a 33G 12 mm hypodermic needle (Meso-relle; UKMedi, UK). Radioactivity was measured via an activimeter (Capintec CRC-15R; Southern Scientific Ltd, UK) and documented immediately prior to injection. All work was performed in line with local rules and UK regulations, the Ionizing Radiations Regulations 2017 (IRR17 plus the Approved Code of Practice) and the Environmental Permitting Regulations 2016 (EPR2016).

**Intravenous injection of radiotracer**

One at a time, an implanted egg was placed in a suitable holder (such as a 35 mm dish) and the window in the eggshell was enlarged using sterile stainless-steel blunt end forceps to increase CAM accessibility for injection of the radiotracer. Whilst enlarging the window, care was taken to avoid dropping eggshell debris inside the egg or removing too much shell, which might damage the CAM and cause subsequent bleeding. A large vein was located by observing the blood flow direction and blood vessel structure. A site near the shell, with the blood flow towards the center of the egg, was chosen for injection ( Figure 5Bi). Injection of air bubbles during the procedure, which may increase the risk of killing the embryo, was avoided by carefully releasing any entrapped air before injection. The pre-loaded 33G needle and 1 mL syringe were inserted into the CAM (first pressure point) next to the chosen vein and then into the vein (second pressure point), ensuring that the vein was accessed from the side to enable visualization of the needle depth and to reduce the chance of puncturing through the blood vessel. Successful insertion of the needle was assessed by carefully moving the needle side-to-side, to check that the blood vessel followed these movements. Once it was confirmed that the needle was in the vein, the radiotracer was injected slowly and continuously in the direction of the blood flow ( Figure 5Bi, Supplementary video 1 can be viewed via the figshare research repository ( Schulze J, 2025)). Finally, the needle was slowly removed. In case of bleeding, light compression with a sterile wipe was applied for 30-60 seconds to stop any outflow ( Figure 5Bii). Bleeding caused by the injection technique did not typically cause issues if it was stopped swiftly. Two eggs were prepared for imaging at a time, with 20-minute intervals between subsequent injections. Radioactivity before and after the injection was recorded and used to calculate the ‘activity of injected dose at time of injection’ (Equation 1-2, Table 7).

**Table 7.** Equations.

Equation		Terms
**(1)**	$\lambda =\frac{\ln(2)}{{\text{Halflife}}_{\mathrm{FDG}}\;T_{1/2}}$	***λ*** = Decay constant (min ^−1^) for [ ^18^F]-FDG, calculated using equation 1 *Halflife*_*FDG*_ *T*_*1/2*_ = 109.77 minutes *A*_*t*_ = Activity (Bq) of injected dose corrected to decay of radiotracer, calculated using equation 2 *A*_*0*_ = Activity (Bq) of injected dose at time of injection *t* = Time difference (minutes) between injection and start of scanning *SUV*_*t*_ = Standardized Uptake Value at *t* *C*_*ROIt*_ = Concentration of [ ^18^F]-FDG (Bq/mL) in region of interest at *t*. Calculated by Vivoquant software *Body weight* = Weight (g) of egg, average of 50 g weight assumed for all eggs
(2)	$A_{t}=A_{0}*e^{-\mathrm{\lambda t}}$	
(3)	${\mathrm{SUV}}_{t}=\frac{C_{{\mathrm{ROI}}_{t}}*\text{Body weight}}{A_{t}}$	

**Technical considerations for intravenous injection**

In principle, IV injections can be performed from E8 or earlier ( Easty *et al.*, 1969). However, commercially available needles with a small enough gauge are difficult to acquire and pulling needles in house requires specialist equipment. Therefore, we recommend performing IV injection from E11 onwards since a higher injection success rate is achievable due to larger blood vessels being available to inject. Choosing a larger vessel reduces the chance of causing damage and blood loss, thus having less effect on survival. Once competent in the radiotracer injection technique, there is the potential to image at least 24 eggs per day, with the only limiting factor being the activity of the decaying radiotracer.

**CT scan followed by static PET (Static PET/CT)**

Eggs were incubated for at least 30 minutes post-injection to allow radiotracer distribution and uptake before scanning ( Warnock *et al.*, 2013, Smith *et al.*, 2023). CT scans were carried out before PET to determine the optimal field of view (FOV, optimization of bed position) to ensure a high spatial resolution. Up to two eggs were placed into the rat bed, held in place with a piece of scotch tape ( Figure 5Biii) and loaded into the X-CUBE Preclinical CT imager (Molecubes NV, Belgium) together ( Figure 5Biv). CT scans were acquired using the standard protocol (‘general purpose’; eggs received 27 mGy dose). Once the CT scans were complete, the multimodal bed containing the eggs was immediately transferred to the β-CUBE Preclinical PET imager (Molecubes NV). Acquisition duration was set to 15 minutes and started in the optimal bed position (as determined via CT). The time between injection of the radiotracer and starting PET imaging was noted. Once scanning was complete, the eggs were returned to the incubator to maintain the correct temperature.

**PET/CT data reconstruction**

**Figure 6.**
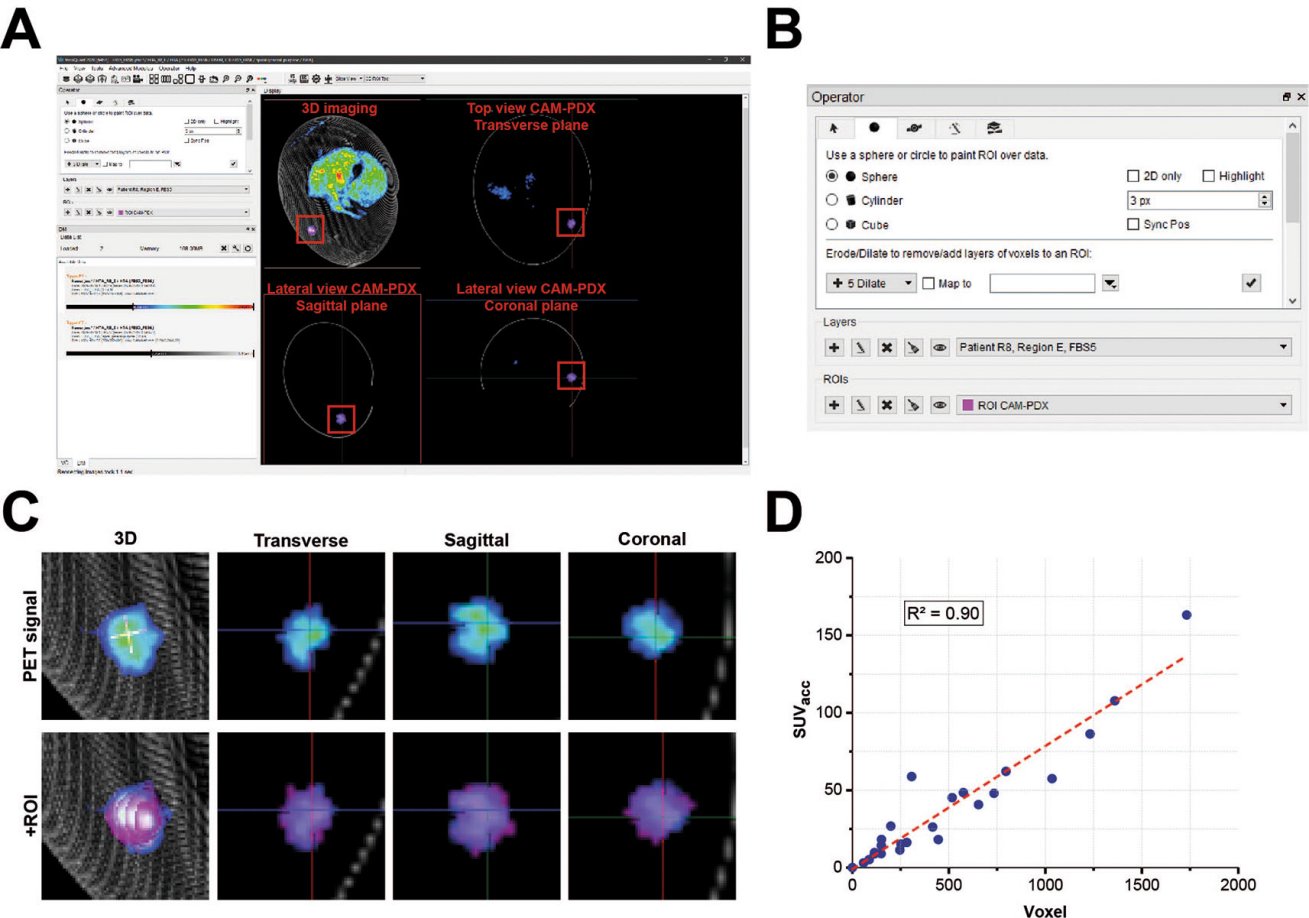
PET data analysis. A, User Interface of Invicro Vivoquant software showing different sectional planes (transverse, sagittal and coronal) from a reconstructed CAM-PDX. B, Input controls and operator settings for drawing the region of interest (ROI) manually (painting tool; sphere, size set to 3 pixel). C, CAM-PDX PET signal of example shown in A overlaid with manually drawn ROI. D, Linearity between voxel size and accumulated standardized uptake value (SUVacc) PET signal for different CAM-PDX underlined consistency in manual ROI drawing. Coefficient of determination; R ^2^ = 0.90. Values used are reported in Table 9.

The PET/CT data were reconstructed (see Table 8 for settings) using the proprietary Molecubes software (Build version: 1.7.6 H 989d0998; Copyright license registered to University of Liverpool – Centre for Preclinical Imaging). Reconstructed PET/CT data were then visualized, images co-registered and PET signal from PDX tumors quantified by using Invicro VivoQuant 2020 (Copyright license registered to University of Liverpool – Centre for Preclinical Imaging; RRID:SCR_025778; Invicro LLCm, MA, USA). A three-dimensional region of interest (ROI) was manually drawn around each xenograft ( Figure 6) to quantify the concentration of [ ^18^F]-FDG at a defined timepoint (C _ROIt_). This value was used to calculate the accumulated standardized uptake value (SUVacc) across the CAM-PDX (Equation 3, Table 7 ( Basu *et al.*, 2007)).

**Table 8.** Positron emission tomography/computed tomography (PET/CT) data reconstruction settings.

**CT data**	Algorithm type: iterative Isometric voxel size: 200 μm Noise reduction: no
**PET data**	Isometric voxel size: 400 μm Number of iterations: 30 Energy resolution: 511 kEV/30%

**Table 9.** Positron emission tomography (PET) data. SUVacc = accumulated standardized uptake value. ROI = region of interest analyzed with size in voxels. The values used to calculate SUVacc can be viewed via the figshare research repository ( Schulze J, 2025).

Experiment	Patient sample ID	Region	Egg ID	Freezing media	SUVacc	ROI (Voxels)
3	R8	E	1	Low FBS	16.238	282
3	R8	E	2	Low FBS	57.468	1035
3	R8	E	3	Low FBS	48.024	734
3	R8	E	7	Low FBS	45.192	517
3	R8	E	5	High FBS	107.710	1360
4	R8	D	2	Low FBS	62.162	796
4	R8	D	3	Low FBS	5.158	87
4	R8	D	1	High FBS	11.375	246
4	R8	D	2	High FBS	26.206	416
4	R8	D	5	High FBS	15.201	252
5	R8	F	1	High FBS	9.009	149
5	R8	F	4	High FBS	86.311	1232
5	R8	F	5	High FBS	40.677	654
5	R8	F	3	Low FBS	163.126	1733
6	R8	B	1	Low FBS	8.848	114
6	R8	B	2	Low FBS	48.462	575
6	R8	B	4	Low FBS	18.168	151
7	R8	A	14	High FBS	58.827	307
7	R8	A	18	High FBS	9.762	115
7	R8	A	21	High FBS	0.000	0
7	R8	A	22	High FBS	14.253	150
7	R8	A	23	High FBS	26.798	199
7	R8	A	24	High FBS	3.047	58
7	R8	A	25	High FBS	18.121	445

**Experimental design and statistical evaluation**

Each xenografted egg was considered as one biological unit and all eggs prepared and imaged together as one experimental unit. Sample size per experimental unit was determined by the number of eggs (24 maximum) that can be scanned in one day. Biological units were included if they underwent successful engraftment and tracer injection. Biological units were excluded if they were unfertilized, died prior to E13 or showed signs of contamination. To account for exclusions, experiments were started with twice the number of biological units required at E14. For comparison of frozen tissue, biological units were randomly divided into two groups on E7.

Statistical analyses were performed in OriginPro 2021b Academic (OriginLab Corporation, MA, USA; RRID:SCR_014212). Distribution and variance of data was assessed by Shapiro–Wilk test and Two-Sample Test for variance, respectively. Data were then analyzed by parametric or non-parametric test as appropriate. The number of independent samples, definition of error bars, and the statistical test employed are described in relevant figure legends. *p* values less than 0.05 were considered significant.

### Controlling bias, variability and limitations of datasets

**Potential sources of bias**

Sample bias: No formal inclusion or exclusion criteria were applied to the sample collection and all samples available from patients that consented were collected without prior knowledge of histological subtype, or BAP1 status ( Table 10). Samples are linked anonymized but patient information, such as age and gender, has not been accessed during method development. However, we would expect a bias towards epithelioid as this represents 70% of mesothelioma cases in England and Wales, as well as samples from older males, reflecting the much higher incidence of mesothelioma in men ( Husain *et al.*, 2024).

**Table 10.** Patient tissue collected under Mesobank ethics. Summary of the patient samples collected indicating our in-house ID, whether it was subsequently confirmed as mesothelioma, histological subtype, BRCA1-associated protein 1 (BAP1) status, whether the sample has been assessed for implantation (CAM-PDX), and which figures the samples appear in. N/A = not applicable.

ID	Mesothelioma diagnosis	Histological subtype	BAP1 status	CAM-PDX	Figures
R1	NO	N/A	N/A	N/A	N/A
R2	YES	Sarcomatoid	Positive	Yes	3, 7
R3	NO	N/A	N/A	N/A	N/A
R4	YES	Epithelioid	Unknown	Yes	N/A
R5	YES	Epithelioid	Negative	Yes	12
R6	NO	N/A	N/A	N/A	N/A
R7	NO	N/A	N/A	N/A	N/A
R8	YES	Epithelioid	Positive	Yes	4, 8, 9, 10, 11, 13
R9	NO	N/A	N/A	N/A	N/A
R10	YES	Epithelioid	Negative	No	N/A
R11	YES	Epithelioid	Positive	Yes	8
R12	NO	N/A	N/A	N/A	N/A
R13	NO	N/A	N/A	N/A	N/A
R16	YES	Epithelioid	Unknown at time of publication	Yes	N/A

Vascularization scoring: To reduce observer bias, each CAM-PDX was scored macroscopically for engraftment by two independent researchers.

PET/CT analysis: To quantify the PET signal for each CAM-PDX, a ROI was manually drawn in the Invicro Vivoquant software ( Figure 6). For consistency the same settings (geometry and size) for the selection tool were used each time. To check for variability in ROIs, the SUVacc for each CAM-PDX was plotted against voxel size. A coefficient of determination (R ^2^) of 0.90 confirmed consistency between ROIs.

**Potential sources of variability**

Egg survival: Many factors influence egg quality and variability in survival, including the climate the chickens are exposed to before laying and sudden changes to their environment, temperature during transportation, duration between laying and initiating development, temperature fluctuations during the experiment, number of interventions during the experiment, cleanliness and decontamination procedures. In our experience survival to E14 can range from 50 to 80%.

Implantation: There could be inter-researcher differences in technique, however experiments were performed by three independent researchers according to standard operating protocols.

PET signal readout: Standardized uptake value (SUV) allows for normalization between CAM-PDXs by removing variability introduced through injected doses and time differences.

**Limitations of datasets**

Patient samples: Since this article is intended to demonstrate proof of principle and methodology development, here we present detailed data for samples collected from only three patients (one sarcomatoid and two epithelioid). However, vascularization of samples from five patients is also summarized. An overview of all patient samples collected is provided in Table 10.

## Results

**Figure 7.**
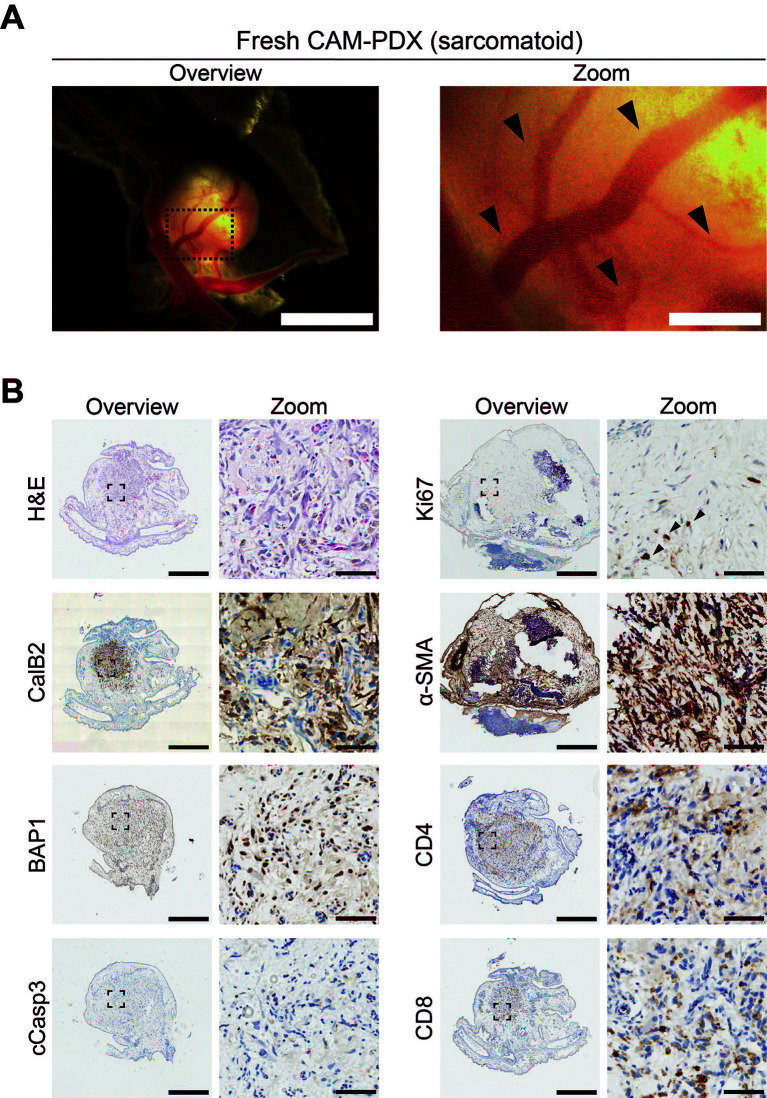
Characterization of a sarcomatoid mesothelioma CAM-PDX generated from a fresh biopsy tissue sample. A, Representative brightfield image of freshly dissected CAM-PDX (sarcomatoid; n = 15) at E14, imaged from underneath to show vascularization. Scalebar in overview image represents 2.5 mm and in adjacent zoom image 500 μm. Black arrowheads highlight CAM-PDX feeder vessels. B, Histological and immunohistochemical staining of CAM-PDX generated from fresh sarcomatoid tissue fragments. Images are for example shown in A and are representative of the following CAM-PDXs: H&E n = 6, CalB2 n = 6, BAP1 n = 1, cCasp3 n = 1, Ki67 n = 5, α-SMA n = 5, CD4 n = 5, CD8 n = 5. A minimum of 3 sections per stain per sample were used. Scalebars in overview images represent 500 μm and in adjacent zoom images 50 μm. Black arrowheads highlight Ki67 positive cells.

As initial proof of principle that freshly collected mesothelioma tissue can engraft onto the CAM, we scheduled initiation of embryo development and windowing so that eggs were ready for engraftment at E7 on the day of the biopsy surgery. We implanted 1 mm ^3^ fragments of fresh sarcomatoid tissue at E7 and, by E14, we observed that 93% (n = 15) of nodules were vascularized based on evidence of remodeling of the CAM vasculature and feeder vessels ( Figures 3 and 7A; Table 11). Immunohistochemical assessment of the PDXs showed the presence of calretinin (CalB2)-positive mesothelioma cells and expert pathological assessment of H&E staining found localized spindle cell proliferation showing atypical cells with hyperchromatic nuclei favoring sarcomatoid mesothelioma cells, which was consistent with the patient’s diagnosis. The mesothelioma cells were BAP1-positive, in line with expectations for the sarcomatoid subtype since only 20% are BAP1 negative ( Righi *et al.*, 2016). Additionally, Ki67-positive proliferating cells were present (black arrowheads, Figure 7B), with low levels of apoptosis as determined by caspase-3 (cCasp3) staining ( Figure 7B). We also observed the presence of host and/or patient-derived alpha smooth muscle actin (α-SMA)-positive fibroblasts, as well as preservation of patient-derived CD8+ and CD4+ immune cells ( Figure 7B).

**Table 11.** CAM-PDX vascularization scoring.

	Vascularization score - n (%)		
	Yes	No	Not scorable
***Fresh CAM-PDX ***			
All (n = 17)	16 (94%)	1 (6%)	0 (0%)
Sarcomatoid (n = 15)	14 (93%)	1 (7%)	0 (0%)
Epithelioid (n = 2)	2 (100%)	0 (0%)	0 (0%)
***Cryopreserved CAM-PDX ***			
All (n = 52)	42 (78%)	6 (11%)	6 (11%)
High FBS (n = 27)	22 (82%)	2 (7%)	3 (11%)
Low FBS (n = 26)	20 (74%)	4 (15%)	2 (11%)

**Figure 8.**
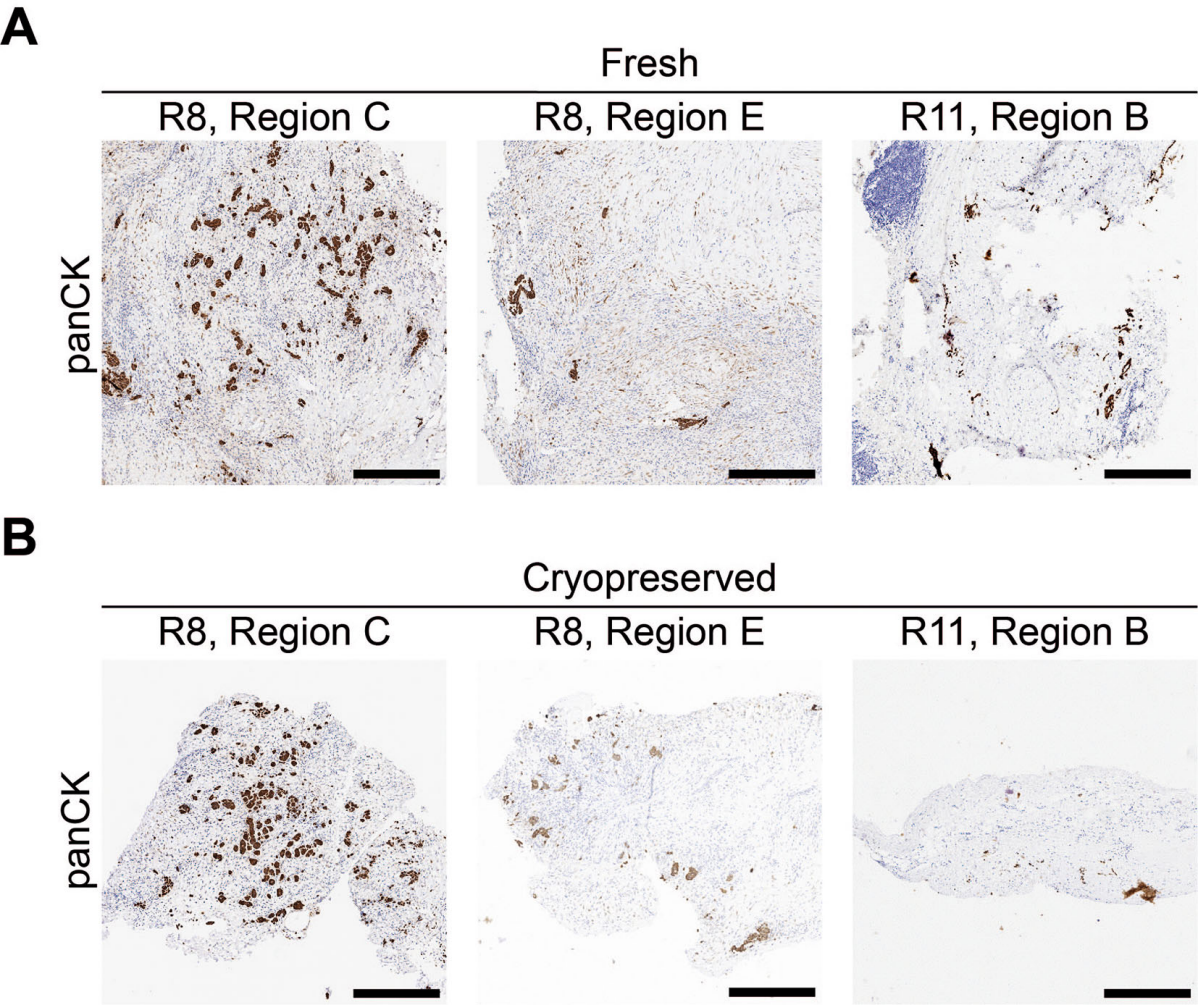
Mesothelioma tumor cells are diffusely dispersed throughout the collected tissue. Example of pan cytokeratin (panCK) staining of mesothelioma cells for three fresh-fixed regions from two patient biopsy samples (A) and three fragments that were cryopreserved prior to fixation from the same three regions (B). Images are representative for the following samples: R8 n = 7, R11 n = 2. A minimum of 3 sections were used per stain, per sample. Scale bars represent 500 μm.

Since pleural mesothelioma is a heterogenous cancer, where tumor cell content and distribution can differ greatly between, and within patient samples ( Figure 8A), a workflow to facilitate pre-screening of the tissue samples ( Figure 1A) was employed to optimize selection for implanting and ensure regions with a greater proportion of tumor cells are prioritized for generating the CAM-PDXs. Moreover, surplus biopsy material was obtained immediately after procedure and only 50% of samples collected were later determined to be mesothelioma by the clinical diagnostic service ( Table 10). Therefore, a freezing protocol was utilized to preserve the tissue whilst maintaining histological concordance with the fresh counterpart ( Figure 8B), to allow time for histopathological confirmation of mesothelioma and, in line with 3Rs principles, prevent egg development being initiated unnecessarily should surgery not go ahead as planned, or implanted samples later be determined as not mesothelioma.

**Figure 9.**
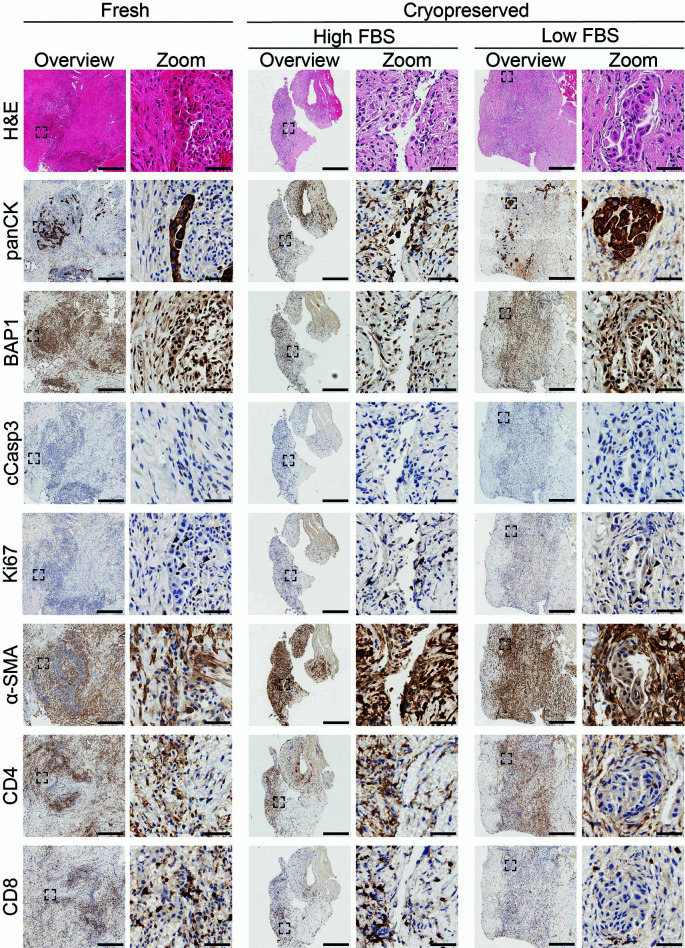
Comparison of fresh-fixed and slow-frozen epithelioid mesothelioma biopsy tissue. Representative histological and immunohistochemical staining of fresh-fixed control tissue and tissue cryopreserved in high or low FBS freezing media. Fresh samples: H&E n = 15, panCK n = 15, BAP1 n = 5, cCasp3 n = 1, Ki67 n = 2, α-SMA n = 1, CD4 n = 1, CD8 n = 1. High FBS: H&E n = 9, panCK n = 10, BAP1 n = 2, cCasp3 n = 1, ki67 n = 2, α-SMA n = 1, CD4 n = 1, CD8 n = 1. Low FBS: H&E n = 7, panCK n = 7, BAP1 n = 2, cCasp3 n = 1, Ki67 n = 2, α-SMA n = 1, CD4 n = 1, CD8 n = 1. A minimum of 3 sections per stain per sample were used. Scalebars in overview images represent 500 μm and in adjacent zoom images 50 μm.

Next, we sought to assess two commonly used freezing media, termed “High FBS” or “Low FBS” ( Table 2), to determine which best preserves the tissue architecture and viability of the collected mesothelioma biopsy material. Adjacent fragments of tissue were slow-frozen in either high or low FBS freezing media with one piece of fresh tissue immediately fixed (“fresh-fixed”) for histology ( Figure 1). After a period (>1 month) in liquid nitrogen storage, exemplar fragments were thawed and fixed for histology. H&E staining revealed standard artifacts from freezing in both cryopreservation media, however viable tumor cells were present as confirmed by a pathologist ( Figure 9). Immunohistochemical staining confirmed the presence of tumor cells (panCK-positive) that histologically matched the patient diagnosis of BAP1-positive epithelioid mesothelioma, across all three conditions ( Figure 9). Further characterization revealed the presence of proliferating cells and no visible apoptosis. Staining for α-SMA showed similar fibroblast composition, whilst CD4+ and CD8+ immune cells were detected in all conditions. Overall, these data show that both freezing methods were successful in preserving tissue architecture whilst maintaining the cellular composition of the sample.

**Figure 10.**
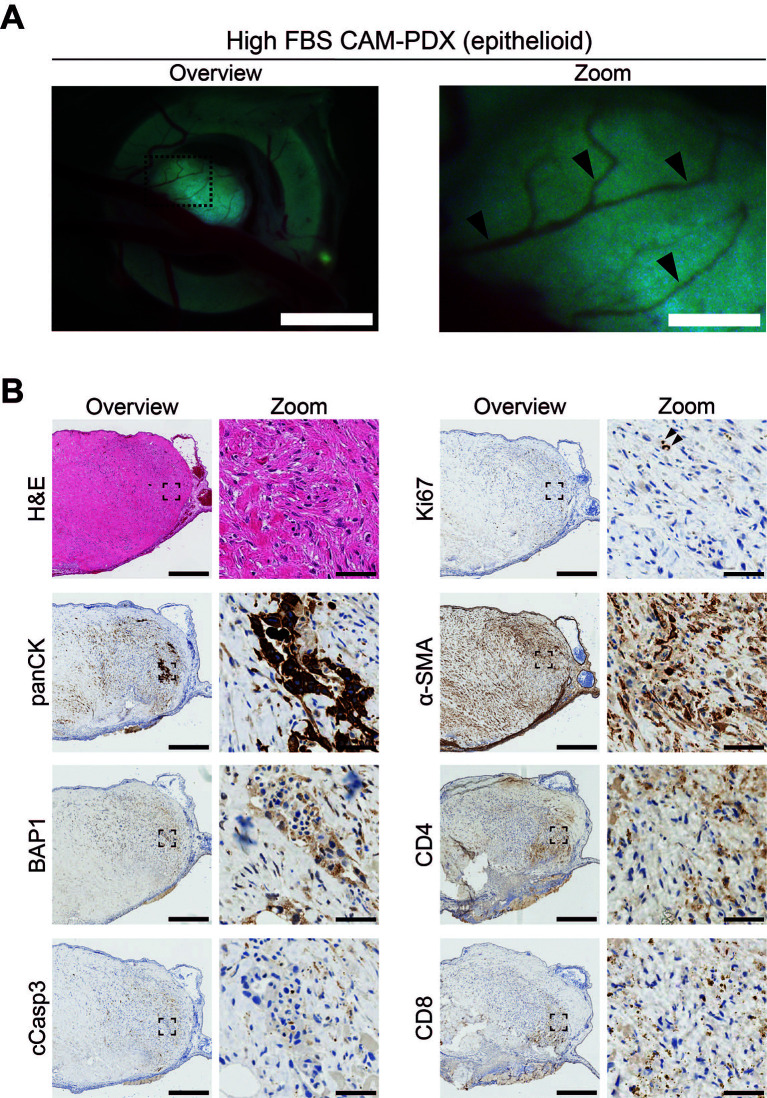
Epithelioid mesothelioma CAM-PDX generated from cryopreserved biopsy tissue fragments. A, Representative brightfield images of freshly dissected CAM-PDX at E14, generated from epithelioid mesothelioma tissue cryopreserved in high FBS freezing media ( Table 2, n = 22 CAM-PDX). CAM-PDX imaged from underneath to highlight vascularization. Scalebars in overview images represent 2.5 mm and 500 μm in adjacent zoom images. B, Histological and immunohistochemical staining of engrafted nodule from cryopreserved tissue. Images are representative of the following CAM-PDXs: H&E n = 15, panCK n = 10, BAP1 n = 5, cCasp3 n = 5, ki67 n = 9, α-SMA n = 5, CD4 n = 2, CD8 n = 2. A minimum of 3 sections per stain, per CAM-PDX were used. Scalebars in overview images represent 500 μm and in adjacent zoom images 50 μm.

**Figure 11.**
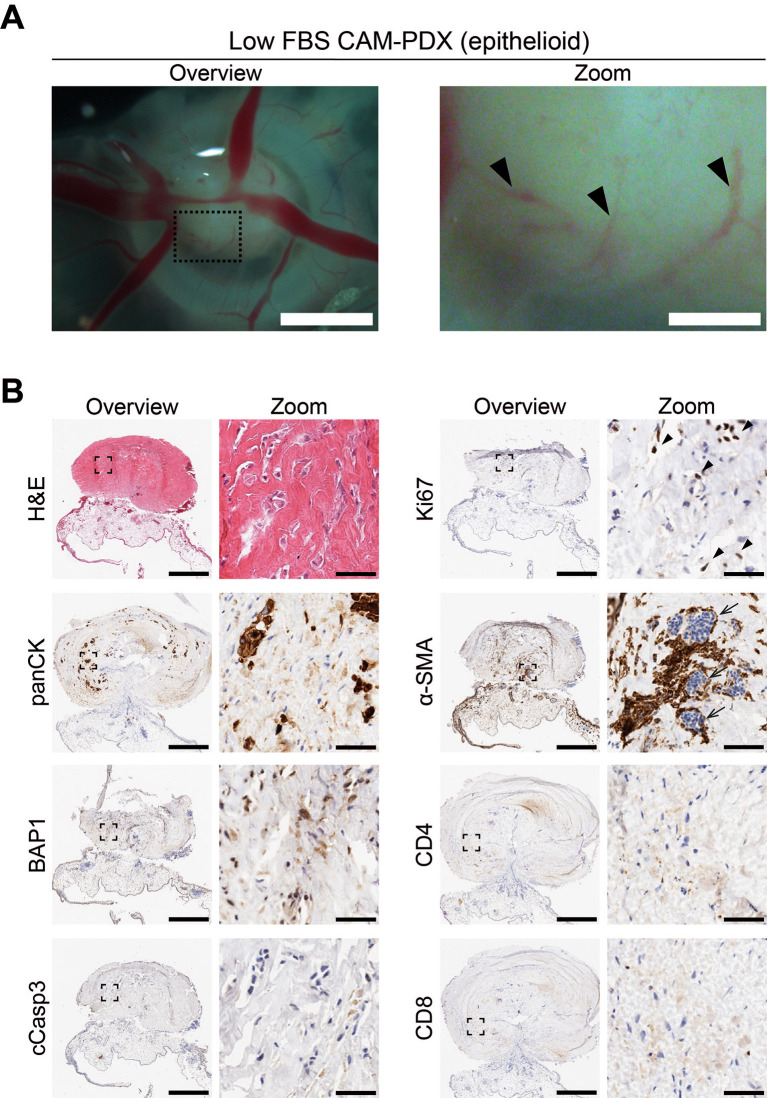
Epithelioid mesothelioma CAM-PDX generated from cryopreserved biopsy tissue fragments. A, Representative brightfield images of freshly dissected CAM-PDX at E14, generated from epithelioid mesothelioma tissue cryopreserved in low FBS freezing medium ( Table 2, n = 20 CAM-PDX). CAM-PDX imaged from underneath to highlight vascularization. Scalebars in overview images represent 2.5 mm and in adjacent zoom images 500 μm. B, Histological and immunohistochemical staining of engrafted nodules from cryopreserved tissue. Images are representative of the following CAM-PDXs: H&E n = 15, panCK n = 10, BAP1 n = 5, cCasp3 n = 5, Ki67 n = 9, α-SMA n = 5, CD4 n = 2, CD8 n = 2. A minimum of 3 sections per stain per sample were used. Scalebars in overview images represent 500 μm and in adjacent zoom images 50 μm (B).

**Figure 12.**
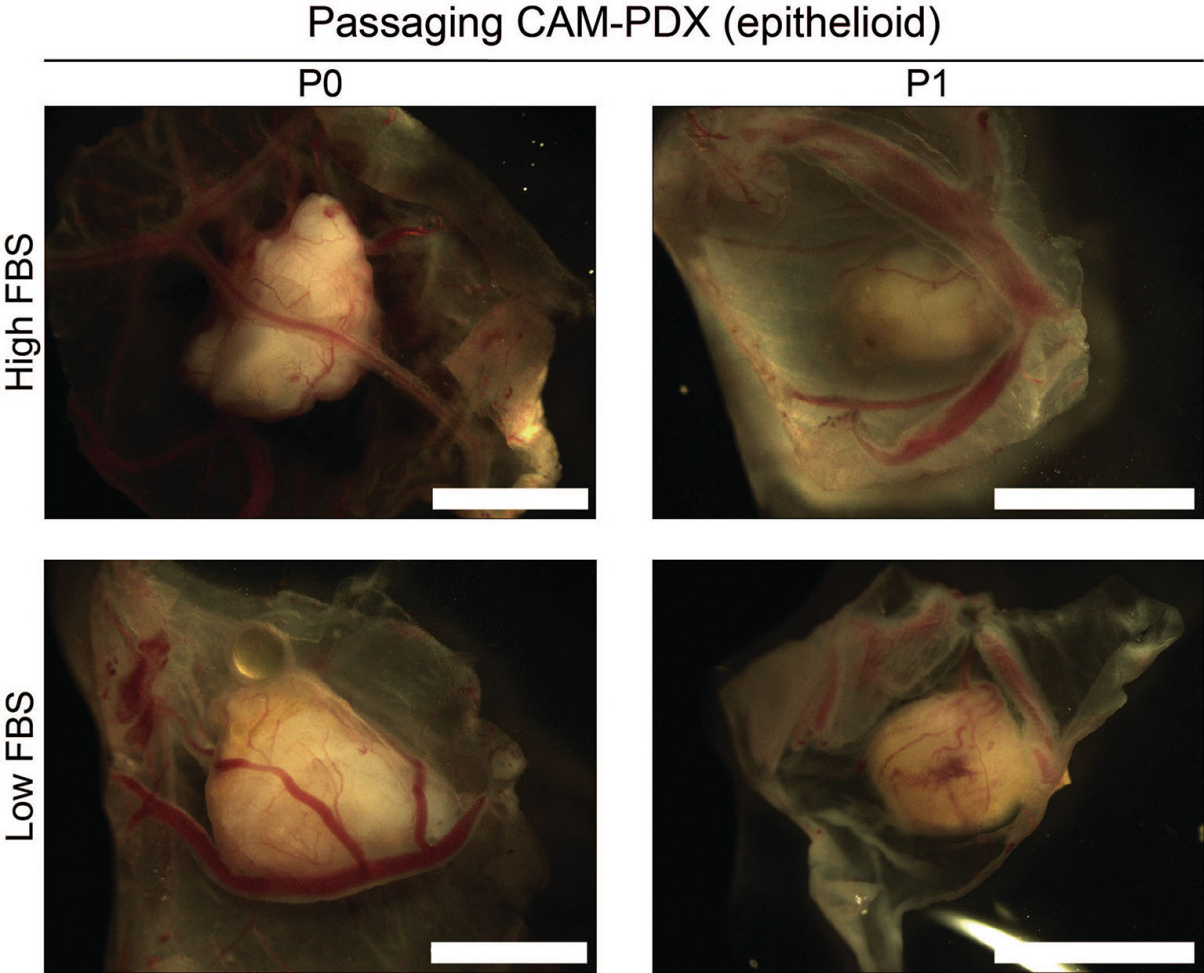
Passaging of CAM-PDX. Representative brightfield images of dissected CAM-PDX at embryonic day 14. Images show CAM-PDX generated from epithelioid mesothelioma tissue cryopreserved in high FBS (top, n = 3) or low FBS (bottom, n = 2) freezing media (P0) and the corresponding passaged CAM-PDX (P1; high FBS n = 2, low FBS n = 2). CAM-PDX imaged from underneath to highlight vascularization. Scale bars represent 2.5 mm.

To assess reanimation of viable tissue, we thawed fragments slow-frozen in high or low FBS freezing media (from the same epithelioid biopsy sample region shown in Figures 8 and 9) and implanted them onto the CAM. Visual scoring for remodeling of the CAM vasculature and feeder vessels showed that 82% (n = 27) versus 74% (n = 26) of PDX generated from tissue fragments stored in high or low FBS freezing media, respectively, had formed well vascularized nodules after 7 days ( Figures 10A and 11A, Table 11). On E14, the resulting CAM-PDXs were dissected and processed for histological assessment. We observed retention of mesothelioma cells and BAP1 status, along with presence of proliferating Ki67 cells (black arrowheads, Figure 11B) and little evidence of apoptosis ( Figures 10B and 11B). There was also evidence on H&E of nucleated red blood cells inside cross sections of vessel-like (α-SMA positive) structures in the CAM-PDXs (white arrows, Figure 11B), indicating perfusion of chick blood within pre-existing or new vasculature within the engrafted tissue. The only discernible difference observed was that the frequency of CD4+ and CD8+ T-cells was lower in the CAM-PDXs derived from tissue frozen in the low FBS media ( Figure 11B). Overall, these data show successful engraftment on the CAM following reanimation after freezing and it should be noted that despite the sparse tumor cell content of the example CAM-PDX shown in Figure 10, it offers proof of principle of the methodology irrespective of how challenging the sample is. Finally, as it is common to serially passage mouse PDX ( Wu *et al.*, 2017), we assessed the feasibility of this for the CAM-PDX model. Briefly, CAM-PDX generated from frozen epithelioid fragments were dissected on E14 (P0), cut in half and transplanted to new E7 eggs (P1). We observed vascularization of the nodules following passaging irrespective of the freezing method ( Figure 12). However, expansion of the tissue was not evident over the 7-day engraftment period, likely reflecting the diffuse tumor cellularity and biology of mesothelioma.

**Figure 13.**
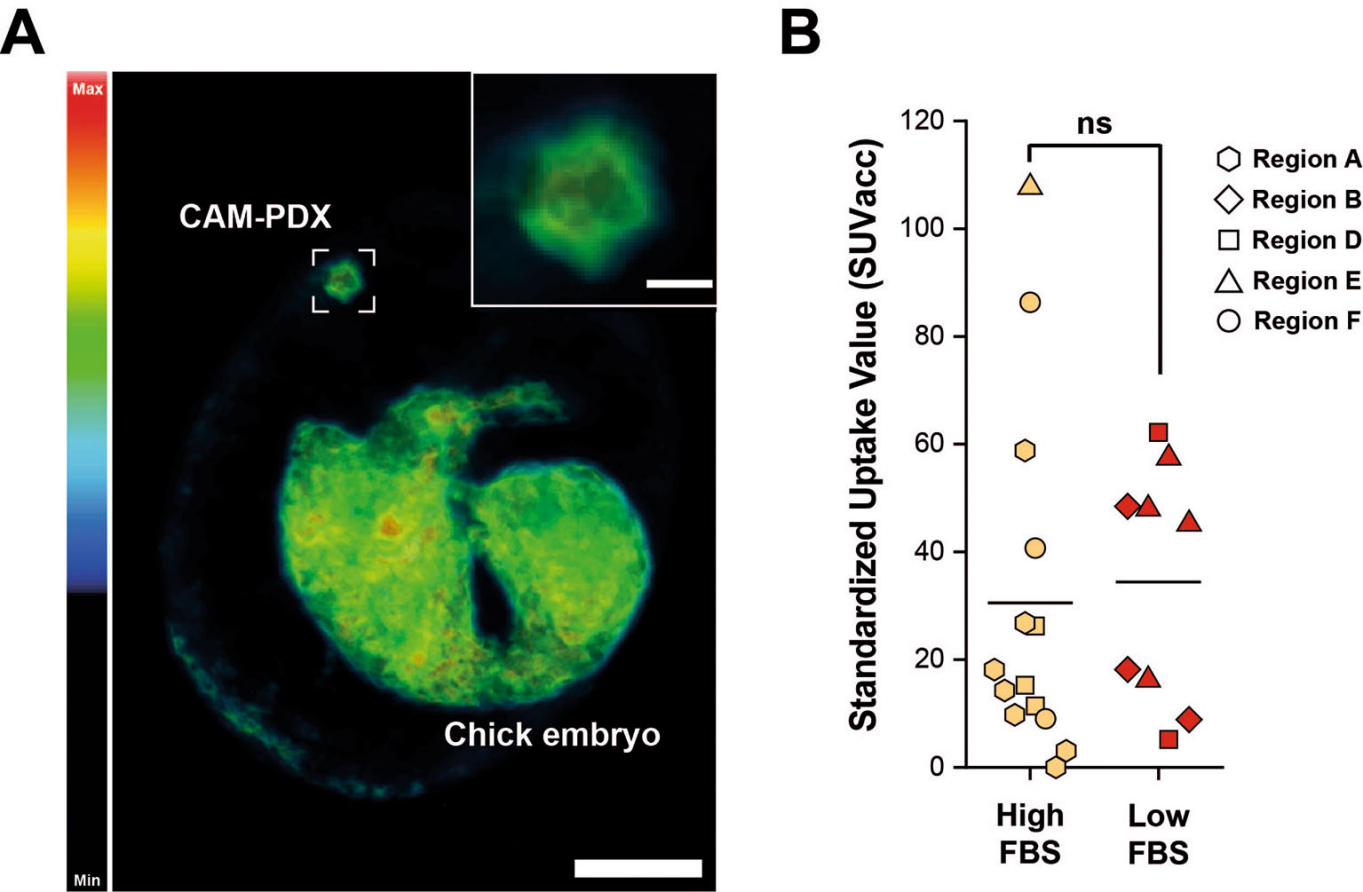
PET/CT imaging of epithelioid mesothelioma CAM-PDX generated from cryopreserved tissue. A, Representative reconstruction of [ ^18^F]-FDG PET/CT imaging. Color scale represents tracer uptake (Bq/mL, Min = 0, Max = 2.5 × 10 ^4^). Scalebar in overview image represents 1 cm and in inset image 2.5 mm. B, PET quantification of [ ^18^F] _-_FDG signal from CAM-PDX indicates no significant difference between fragments cryopreserved in high FBS (mean ± SD = 30.52 ± 32.35; n=14) or low FBS (mean ± SD = 34.41 ± 22.09; n = 9) media. Line represents population mean, Two Sample t-test, p=0.76, not significant (ns). Data was acquired from 5 independent experiments. Sample information and SUVacc values provided in Table 9.

To visualize viable mesothelioma cells within the CAM-PDXs *in ovo*, we required an imaging modality that is suitable for unlabeled tissue and we therefore utilized [ ^18^F]-FDG PET/CT. [ ^18^F]-FDG is a non-metabolizable glucose analogue widely used as a radiotracer in oncology, including mesothelioma ( Szlosarek *et al.*, 2013), as elevated glucose uptake is commonly observed in cancer cells due to the Warburg effect ( Tekade and Sun, 2017). PET imaging after intravenous administration of [ ^18^F]-FDG revealed tracer uptake ( Figure 13A) in the CAM-PDX which were established from the same cryopreserved epithelioid tissue shown in Figures 8- 11. [ ^18^F]-FDG uptake indicates that the CAM-PDXs were sufficiently well vascularized to accumulate administered substances, such as the radiotracer, from circulating blood, and that they are metabolically active. Quantification of the PET signal, reported as total accumulated SUV across the entire CAM-PDX (SUVacc), showed similar uptake in CAM-PDXs (Two Sample t-test, p = 0.76), irrespective of the cryopreservation media used ( Figure 13B), suggesting that both freezing methods preserve viable tissue that can be reanimated for engraftment. Despite this, we recommend high FBS freezing media, as a higher percentage of nodules became vascularized and there is evidence of better resident immune cell preservation.

## Use cases

The CAM assay is a cost-effective, higher throughput preclinical model that can contribute to accelerating translation of *in vitro* studies and the treatment discovery pipeline. With increased uptake of the CAM model in the research community, it will become more widely accepted as a direct replacement for the ‘gold standard’ mouse model, therefore having a greater contribution to reducing the use of mammalian models. We propose that as the model is moderate throughput, drug evaluation can be conducted on first generation (P0) CAM-PDXs, potentially further reducing numbers, as it would remove the standard practice of serial passaging in mice. If one successful mouse PDX is typically passaged 5-times, using 5 mice each time, then the CAM-PDX model could theoretically reduce the number of animals used per PDX by 25. We expect the CAM-PDX model to be utilized for applications such testing combination therapies, formulations, drug delivery systems and novel treatment strategies. To ensure experimental rigor, we recommend that the study design should incorporate the appropriate controls such as blinding to any information relating to the sample and treatment condition, for all stages of experimental intervention, imaging and analysis.

## Conclusions/Discussion

We have shown that viable, vascularized mesothelioma CAM-PDXs can be generated from both fresh and frozen fragments of biopsy tissue, and that these are amenable to viability assessment using PET/CT imaging. Importantly, morphological and immunological features of the primary sample are retained, the latter being a key advantage over cell line-derived CAM xenografts and methods that involve disaggregation of patient derived material, particularly with the growing importance of immunotherapies. The inclusion of PET/CT readout is highly translational and relevant, since it is routinely used in the clinic to detect and stage cancer as well as monitor treatment response. Together the methods we describe will allow assessment of response to a broad range of therapies in an ethical model that provides an excellent recapitulation of human disease and allows systemic administration of drugs.

We have established the feasibility of cryopreserving tissue prior to PDX generation bringing together several practical and 3Rs benefits, making these models more readily adoptable. For instance, when utilizing surplus material from diagnostic biopsies not all will be confirmed as cancer, indeed in our case only half of samples we collected were confirmed as mesothelioma. Careful tissue processing and cryopreservation enables histopathological confirmation of the cancer type prior to starting an experiment. Furthermore due to the heterogeneous nature of pleural mesothelioma, immunohistochemical pre-screening of the tissue to assess tumor cell content increases the chances of using tumor-cell rich regions. Although CAM xenograft models are non-protected up to embryonic day 14 ( The Animals (Scientific Procedures) Act 1986 Amendment Regulations, 2012) and more ethical than mouse xenograft models ( Ribatti and Annese, 2023), they are not truly animal-free. Therefore, the methodology adaptations we describe here are important to ensure that embryo development is only initiated when good quality and well characterized banked tissue is available for PDX engraftment.

Our work is also applicable across other mesothelioma models that utilize patient tissue. In an era when therapeutic surgery is increasingly rare following publication of the MARS2 study ( Lim *et al.*, 2024), the tissue pre-screening workflow described here can be utilized for efficient generation of any patient-derived models from biopsy tissue, including *in vitro* PDEs. In addition, where mouse PDX models are necessary, animal numbers can be reduced by ensuring only tissue confirmed as the cancer of interest, as well as tumor cell-rich regions are utilized. Freezing samples prior to engraftment also has the benefit of widening the availability of tissue, and therefore the appeal of the CAM-PDX model, to researchers who may be unable to readily obtain fresh tissue, for example due to a lack of local specialist hospitals or clinical collaborators.

The CAM-PDX model sits between PDEs and mouse PDXs, offering a middle-ground between tractability, time scale and physiological relevance. A particular advantage over *in vitro* methods is host-derived vascularization that is only achievable with *in vivo* models. Moreover, the short experimental window of the CAM-PDX model means results can be available in weeks compared to the months required to obtain meaningful data from mouse PDX models. However, since there is only a 7-day window from engraftment to termination of the experiment using the CAM, animal models may still be required for longer term studies, for example if therapies require a prolonged treatment regime. Although the ability to maintain mouse PDX models for long periods does have drawbacks, for instance it allows more time for the human tissue to be infiltrated by host-derived cells. It is also common practice to passage mouse xenografts to extend study time and expand material ( Wu *et al.*, 2017). However, serial passaging can result in loss of human-derived stroma ( Julien *et al.*, 2012), immune cells and contribute to further colonization by mouse stromal components leading to a far less representative model over time ( Rosfjord *et al.*, 2014). Although we have shown transplanting CAM-PDXs is possible, there was no evidence for expansion of the material and given the limitations observed with passaging PDX in mice, researchers should carefully consider whether this is necessary.

Here we describe in detail how to perform PET/CT to facilitate *in ovo* assessment of unlabeled xenografts. Whilst PET allows us to visualize the xenografts, CT scans enable the precise localization of the region of interest. It also serves to apply attenuation correction in the PET images, which increases the accuracy of the quantification of radiotracer uptake. Although dynamic scanning allows detailed analysis of physiological processes, it is time-consuming and requires complex data processing. Combining static PET imaging with an efficient injection technique means up to 24 eggs can be scanned per day (8 working hours), only limited by the activity of the decaying radiotracer, making it a high-throughput preclinical screening tool.

The experimental pipeline we describe has the potential to significantly accelerate the advancement of personalized medicine, as it could be utilized as a rapid avatar for treatment selection or establishing patient suitability for clinical trials ( Izumchenko *et al.*, 2017), as opposed to the slower, more costly and less ethical murine PDX models. The option to test treatments in a rapid manner using the CAM-PDX platform, where turnaround time can be weeks instead of months, is highly desirable for cancers with poor prognosis such as mesothelioma, where patients may only survive a few months after their diagnosis.

We believe that these combined protocols for efficient tissue processing and the generation of CAM xenografts from mesothelioma tissue can significantly reduce requirements for mouse PDX models. Furthermore, due to the success rate of generating CAM-PDX from a range of cancers ( DeBord *et al.*, 2018) we envisage this model accommodating most solid cancer types, bringing wider impact in the reduction and replacement of murine models in cancer research. Additionally, due to the minimal requirement for expensive reagents or equipment, the CAM model can be easily adopted in standard research labs. As well as expanding to other cancer types, there is also scope for combining with other preclinical imaging such as magnetic resonance imaging ( Barnett *et al.*, 2022), adding alternative read-outs such as spatial profiling, or volatile organic compound analysis ( Little *et al.*, 2024), and investigating novel therapies ( Schulze *et al.*, 2023b) and delivery vehicles ( Butler *et al.*, 2022). Overall, the need for more accessible and physiological models of cancer, faster translation of therapies and the desire to reduce rodent model usage, makes the CAM-PDX model an attractive option.

## Ethical considerations

The collection and use of human tissue samples for this study was covered by the generic ethical approval granted to Mesobank – a Research Tissue Bank, approved by East of England - Cambridge Central Research Ethics Committee (approved 9 ^th^ August 2019, 18/EE/0161; renewed 30 ^th^ August 2023, 23/EE/0139). Therefore, a separate REC approval for this project was not required. Patient samples were donated under written informed consent and collected between March 2021 and November 2024. All experiments using human tissue were conducted in accordance with the Declaration of Helsinki and in compliance with all local policies and standard operating procedures for working with human material. Samples stored and research performed at the University of Liverpool under licensing no. 12020 granted under section 16(2)(e)(ii) of the Human Tissue Act 2003.

All experiments utilizing fertilized Hens’ eggs were terminated by embryonic day 14 (two thirds of the gestation period) meaning the model is classified as non-protected under The Animals (Scientific Procedures) Act 1986 Amendment Regulations 2012 in the UK and therefore no Home Office approval was required. Standard operating procedures were reviewed and approved by the University of Liverpool Animal Welfare and Ethical Review Body.

## Data Availability

The authors confirm that the data supporting this study are available within the article or via FigShare. FigShare: Methodology for generating chorioallantoic membrane patient-derived xenograft (CAM-PDX) models of pleural mesothelioma and performing preclinical imaging for the translation of cancer studies and drug screening, https://doi.org/10.6084/m9.figshare.28659185.v3 ( Schulze *et al.*, 2025). This contains the following:
- Original CAM-PDX dissection images (.tiff ) - Original histology and immunohistochemistry images (.tiff ) - Supplementary movie 1 (.mov) - 3D rendering of in ovo [ ^18^F]-FDG PET shown in Figure 13A (.gif ) - Raw data used to calculate accumulated standardized uptake values in Figure 13A (.xslx) - Raw PET and CT acquisition files relating to Figure 13B (.DCM) Data are available under the terms of the Creative Commons Attribution 4.0 International license (CC-BY 4.0)
