# Supplementary material for: Methodology for generating chorioallantoic membrane patient-derived xenograft (CAM-PDX) models of pleural mesothelioma and performing preclinical imaging for the translation of cancer studies and drug screening
Source: F1000Res. 2025 Aug 14;14:481. Originally published 2025 May 7. [Version 2] doi: 10.12688/f1000research.163596.2 (PMC12188182; doi:10.12688/f1000research.163596.2)
Supplement: Peer review history

# Reviewer report: Reviewer response for version 1

**DOI:** 10.5256/f1000research.179975.r386170

**Published:** 2025-06-24

**Authors:** Stuart A. Clayton

**Affiliations:** 1 West Virginia University, Morgantown, USA

This article from Schulze et al. demonstrates a novel method for producing mesothelioma patient-derived xenografts utilizing the chorioallantoic membrane of fertilized hen eggs. The CAM-PDX model may also be used with other types of cancer. The article describes the processing of patient tissue, validation of concordance between the original tissue and the passaged tissue, implantation of the tissue sample, and imaging of the CAM-PDX using PET/CT. Overall it is a thorough and well written article that offers an alternative approach to utilizing other PDX models in translational research while reducing the reliance on mammalian animal models.

*Is the rationale for developing the new method (or application) clearly explained?*

**YES**

 The primary rationale behind developing the CAM-PDX method is the potential to reduce animal usage in cancer studies and expedite the personalized medicine approach to drug screening, without losing the translational capability of similar models.

 A small grammatical note in the Introduction (first paragraph, line 3), BRAC1 is written instead of BRCA1, as it’s written elsewhere in the article.

*Is the description of the method technically sound?*

**YES**

 Each section provides adequate detail to explain the methods.

 Verified all catalog numbers correctly correspond with the listed items in Table 1.

 Several sections use the term “sterilization” where the term “disinfection” is more technically accurate. Specifically, the “Preparation of tissue samples for implantation, freezing or fixation for histology” section (page 7, line 2 of that section); the “Implantation” section (page 9, lines 1 and 2 of that section). UV light is usually not sterilizing and ethanol is never sterilizing since they do not effectively kill endospores. Suggest changing to “disinfection” or offering alternative methods for sterilization.

*Are sufficient details provided to allow replication of the method development and its use by others?*

**YES**

 Sections are logically partitioned and easy to follow, and all necessary sections are provided.

 Several sections could be improved by providing further images/diagrams or if possible videos of key steps in the egg preparation and PDX implantation process. Many researchers are likely unfamiliar with using eggs and further visualization may help adoption of the protocol. Specifically, the “Windowing” and “Implantation” sections on page 9. These seem to be vital steps to actually carrying out the protocol and growing the PDXs. Brief videos covering these sections would be helpful to new researchers and they could be placed in the Figshare.

*If any results are presented, are all the source data underlying the results available to ensure full reproducibility?*

**YES**

 The figshare repository is described as containing supplementary data, and I confirmed that the data presented in the paper is actually available there. Additionally, all figures presented in the paper match the raw images in the Figshare repo.

*Are the conclusions about the method and its performance adequately supported by the findings presented in the article?*

**YES**

 The article provides data in support of the feasibility of the method and successful recapitulation of the mesothelioma samples where needed. But in a broader sense it demonstrates the use of the CAM-PDX system in studying cancers while maintaining similarity to the original patient tissue. I agree with the authors conclusions that the CAM-PDX system is a viable alternative to cell or animal models in studying some aspects of cancer.

 A suggestion for further validation of the model, if not already completed in other research articles, would be whole exome sequencing of the original patient tissue and passaged PDX to verify the retention of any key mutations which may be of particular importance when screening drugs and maintaining the personalized approach.

Is the rationale for developing the new method (or application) clearly explained?

Yes

Is the description of the method technically sound?

Yes

Are the conclusions about the method and its performance adequately supported by the findings presented in the article?

Yes

If any results are presented, are all the source data underlying the results available to ensure full reproducibility?

Yes

Are sufficient details provided to allow replication of the method development and its use by others?

Yes

Reviewer Expertise:

breast cancer, patient-derived xenografts, skeletal muscle, bioinformatics. 6+ years of experience working with cancer PDX animal models and performing pre-clinical imaging on them.

I confirm that I have read this submission and believe that I have an appropriate level of expertise to confirm that it is of an acceptable scientific standard.

# Reviewer report: Reviewer response for version 1

**DOI:** 10.5256/f1000research.179975.r386165

**Published:** 2025-06-24

**Authors:** Ulrich Jarry

**Affiliations:** 1 Unité de Pharmacologie Préclinique, Rennes, France

The manuscript entitled " Methodology for generating chorioallantoic membrane patient-derived xenograft (CAM-PDX) models of pleural mesothelioma and performing preclinical imaging for the translation of cancer studies and drug screening" written by Schulze and and his colleagues is a study that describes the procedure to generated a CAM-PDX from mesothelioma.

 The manuscript is well written and really really nice to follow.

 I have no major but some minor points to discuss:

 Introduction section – The authors state that 40 % of mouse experiments are carried out in non-GMO animals, such as xenografts. Personally, I think that many xenograft trials, i.e. transplanting human tumor cells into mice, are carried out in immunodeficient mice such as NSG mice, which are GMOs. I think the sentence needs to be revised.

 The authors specify that the model features immune cells. However, I would caution that the immune system is not functional and that the CAM-PDX model cannot be used to evaluate the efficacy of immunotherapy approaches.

 Material detailed in table 1. RPMI 1640 glutamax is indicated as being from Gibco, while other Gibco products are indicated as being from Fisher Scientific. Please harmonize identification.

 CAM-PDX scoring. Do you have the scoring criteria's ?

Is the rationale for developing the new method (or application) clearly explained?

Yes

Is the description of the method technically sound?

Yes

Are the conclusions about the method and its performance adequately supported by the findings presented in the article?

Yes

If any results are presented, are all the source data underlying the results available to ensure full reproducibility?

Yes

Are sufficient details provided to allow replication of the method development and its use by others?

Yes

Reviewer Expertise:

Preclinical mouse model for oncology and immunology

I confirm that I have read this submission and believe that I have an appropriate level of expertise to confirm that it is of an acceptable scientific standard.
